# Comparative analysis of neural transcriptomes and functional implication of unannotated intronic expression

**DOI:** 10.1186/1471-2164-12-494

**Published:** 2011-10-10

**Authors:** Yazhou Sun, Yaqiong Wang, Yi Hu, Gong Chen, Hong Ma

**Affiliations:** 1 Department of Biology, Pennsylvania State University, University Park, PA 16802, USA; 2 State Key Laboratory of Genetic Engineering and Institute of Plant Biology, School of Life Sciences, Fudan University, Shanghai 200433, P. R. China; 3 The Huck Institutes of the Life Sciences, Pennsylvania State University, University Park, PA 16802, USA; 4 Intercollege Graduate Program in Genetics, Pennsylvania State University, University Park, PA 16802, USA; 5 Institutes of Biomedical Sciences, Fudan University, Shanghai 200032, P. R. China

**Keywords:** neural transcriptomes, stem cell, intronic expression, embryonic brain cortex, neonatal brain cortex

## Abstract

**Background:**

The transcriptome and its regulation bridge the genome and the phenome. Recent RNA-seq studies unveiled complex transcriptomes with previously unknown transcripts and functions. To investigate the characteristics of neural transcriptomes and possible functions of previously unknown transcripts, we analyzed and compared nine recent RNA-seq datasets corresponding to tissues/organs ranging from stem cell, embryonic brain cortex to adult whole brain.

**Results:**

We found that the neural and stem cell transcriptomes share global similarity in both gene and chromosomal expression, but are quite different from those of liver or muscle. We also found an unusually high level of unannotated expression in mouse embryonic brains. The intronic unannotated expression was found to be strongly associated with genes annotated for neurogenesis, axon guidance, negative regulation of transcription, and neural transmission. These functions are the hallmarks of the late embryonic stage cortex, and crucial for synaptogenesis and neural circuit formation.

**Conclusions:**

Our results revealed unique global and local landscapes of neural transcriptomes. It also suggested potential functional roles for previously unknown transcripts actively expressed in the developing brain cortex. Our findings provide new insights into potentially novel genes, gene functions and regulatory mechanisms in early brain development.

## Background

It is well known that total gene numbers are similar among multicellular eukaryotes, and genome size does not correlate with organism complexity, which differs greatly in terms of development, physiology and behavior among eukaryotes [1]. The transcriptome and its regulation contribute significantly to eukaryotic diversity in the aforementioned complexity. The Functional Annotation of the Transcriptome of Mammalian Genome (FANTOM) projects (FANTOM 1-4) have demonstrated the complexity of transcriptomes in several aspects, including non-coding RNAs [2], antisense transcription [2,3], regulated retrotransposon expression [4], and alternative promoter usage, splicing and polyadenylation [5].

Recent high-throughput RNA-seq [6] technologies have provided unprecedented capability to analyze cellular, tissue-specific, or organismal gene activities across a broad spectrum. It also revealed the transcriptomic complexity during cell differentiation [7,8] and organ development [9]. Furthermore, individuals of the same species have transcriptomic differences such as expression variation among humans [10]. Another level of transcriptomic complexities has been revealed by extensive analysis of novel splicing variants from known exons [7-11]. In addition, thousands of transcripts from previously unannotated (non-exonic) genomic regions have been reported [7,8,10-13]; they are either named TUF (Transcripts of Unknown Function) [14] or unannotated TAR (Transcriptionally Active Region) [15]. Some of the unannotated TARs are large intergenic noncoding RNAs that function in embryonic stem cell pluripotency and cell proliferation [16,17], while most unannotated TARs have no known function.

It has been reported that undifferentiated human stem cells have elevated expression of unannotated TARs compared with differentiated neural progenitor cells [7]. Our recent study has also detected additional transcripts from intergenic regions and introns in mouse embryonic and neonatal brain cortices [9]. Mammalian neural development is a complex process involving cell division, cell differentiation, cell migration, axon guidance, synaptogenesis, and synaptic plasticity. The characterization of stage specific unannotated TARs during early brain development could provide clues regarding the roles these unannotated TARs might play in determining neural fate and in regulating neuronal functions.

To further investigate the transcriptome dynamics and to better understand the possible roles of unannotated TARs in early neural development, we have analyzed the RNA-seq datasets from embryonic and postnatal mouse brain cortices that we generated recently [9], as well as seven additional RNA-seq datasets covering both neural and non-neural tissues [7,18]. These nine transcriptome datasets include data from human embryonic stem cell (hESC) and its subsequently differentiated forms (N1, early initiation; N2, neural progenitor; and N3, early glial-like cell) [7], embryonic day 18 (E18) and postnatal day 7 (P7) mouse brain cortices [9], and adult mouse brain (AMB), liver (AML), and muscle (AMM) [18].

Through a systematic analysis of these nine datasets, we found several unique characteristics of the transcriptomes in early neural development. We found that, although the genome was not as pervasively transcribed as previously reported [19], most of the genomic regions at 1 Mb resolution had detectable RNA-seq signals. We also found that the transcriptomes from neural tissues possessed several genome-wide characteristics resembling those of stem cells. Interestingly, the E18 cortex shows the highest level of unannotated transcript expression compared to P7 and adult brains. Furthermore, the intronic unannotated transcripts are associated with GO terms for neurogenesis, neural signaling and negative regulation. Importantly, few of the unannotated TARs in E18 and P7 cortices are connected with known transcripts, suggesting potential novel functions of these TARs during brain development.

## Results and discussion

### Mapping RNA-seq data from mouse developing brains and other organs

To examine the genomic distribution of transcriptomic reads, we mapped all RNA-seq data by the TopHat software [20], which was designed to map RNA-seq data with moderate IT (information technology) infrastructure. Embryonic, neonatal and adult mouse data were mapped onto the mouse reference genome (UCSC mm9, NCBI build 37) as described in the Methods section. For comparison, adult mouse liver and muscle RNA-seq data were analyzed in the same manner. Human ESC and its differentiation data were mapped onto the human reference genome (UCSC hg19, NCBI build 37). Only uniquely matched reads were further analyzed. Because the amounts of data available for the downstream analysis varied for different tissues (Table 1), the read count data were first normalized against the available dataset size, measured in base pairs, for each tissue. To accommodate differences in the details of library preparation and sequencing procedures, we adjusted for the sequencing quality in all data sets according to the quality computation method of the Illumina 1.3 pipeline. E18 and P7 RNA-seq data had the largest percentage of mappable reads in this group, approximately 60%, while other data sets had about 30% mappable data. The AMB RNA-seq data had the largest quantity, with more than 1,000 million mapped base pairs.

**Table 1:** RNA-seq mapping result using TopHat.

	hESC^1^ human embryonic stem cell	hN1^1^ early hESC initiation	hN2^1^ human neural progenitor	hN3^1^ human early glial-like	E18 mouse brain cortex at E18	P7 mouse brain cortex at P7	Adult brain^2^ mouse	Adult liver^2^ mouse	Adult muscle^2^ mouse
Sequencing Platform	Illumina								

Accession Number	SRR037165 to SRR037170	SRR037193 to SRR037198	SRR037199 to SRR037205	SRR037220 to SRR037226	SRP007262	SRP007262	SRR001356 SRR001357 SRR006488 and SRR006489	SRR001358 SRR001359 SRR006490 and SRR006491	SRR001361 SRR001362 and SRR006492

Read Type	35 bp PE and 35 bp SE				36 bp PE and 36 bp SE		33 bp SE		

Original Read Count	14.4 M PE and 4.4 M SE	15.8 M PE and 7.0 M SE	19.6 M PE and 11.6 M SE	22.4 M PE and 3.0 M PE	10.0 M PE and 3.0 M SE	10.4 M PE and 3.6 M SE	89.0 M SE	75.9 M SE	59.9 M SE

Original RNA-seq data size (Mbp)	1,162	1,352	1,774	1,819	853	857	2,936	2,503	1,977

Mapped by Tophat* (Mbp)	401	458	628	594	491	525	1,086	683	571

Percentage Mapped (%)	35	34	35	33	58	61	37	27	29

* Allowing 1 hit by TopHat

1. Data from Wu *et al. *[7].

2. Data from Mortazavi *et al. *[18].

### Most 1-Mb genomic regions have transcriptional activity with uneven distribution at a finer scale

**Figure 1:**
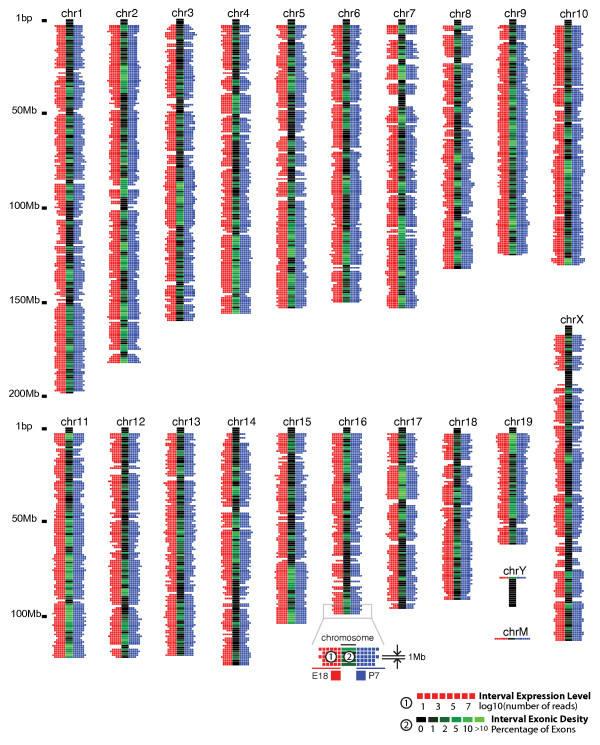
**Genome-wide expression map at 1 Mb resolution for E18 and P7**. Each 1 Mb interval is represented by a horizontal box, with color ranging from black to green. A box with black color means that there was no exon in this given interval. The brighter the green color, the higher the exon content (percentage) this given interval possesses. The scale at the bottom of the map illustrates color representation of the exon percentage of the region. Red square at the left side of the chromosome represents the detected number of RNA-seq reads for the given region in E18 stage, while the blue square at the right side represents the detected number of RNA-seq reads in P7 stage. Expression level is data size normalized. Scale for expression level is at the bottom of the map.

Despite a dataset size up to 1,000 Mbp (AMB), only about 2% of the mouse genome had been mapped with RNA-seq reads, unlike a previous report that suggested a more pervasively transcribed genome from detailed analyses of ~1% of the human genome [19]. A recent study using single- and paired-end RNA-seq and tiling arrays also concluded that the genome is not as pervasively transcribed as previously reported [12]. Nevertheless, when the mouse genome was divided into 1 million base-pair long intervals, we observed that 85% of the intervals had 100 or more detected RNA-seq reads (Figure 1). However, the majority of chromosome regions of the 100 kb size had no detected transcription, as illustrated for X-chromosome (Additional file 1, Fig. S1). Whereas many regions showed transcription at both the E18 and P7 stages, some regions were specifically active in one of these stages, suggesting that a selective set of genes are turned on and off from embryonic to neonatal brain stages, consistent with the previous finding of preferential expression of several thousand genes at either one of these stages [9]. Besides heterochromatin regions (centromeres and telomeres), some large genomic regions with very low annotated gene content had no detectable reads, including several regions in chromosome 7 (Figure 1) and the region in chromosome × from 26,000,000 to 32,000,000 bp (Additional file 1, Fig. S1).

**Figure 2:**
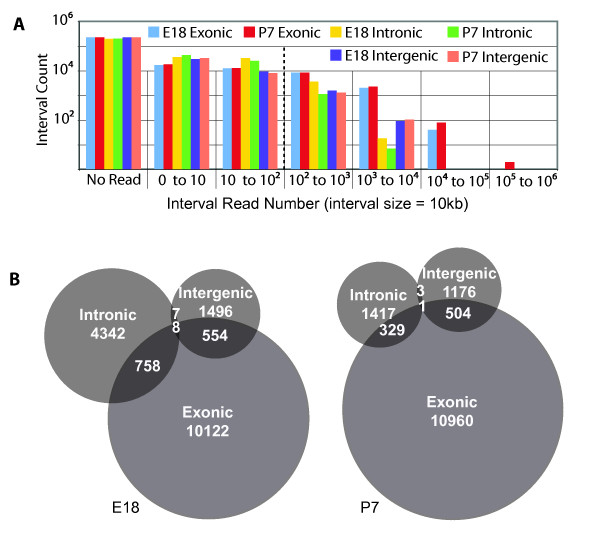
**Genomic expression analysis in 10 Kb interval**. A. Number of intervals in different categories of RNA-seq read coverage. RNA-seq reads were categorized as: *exonic*, which were reads mapped to known exon, *intronic*, which were reads mapped to known intron, and *intergenic*, which were reads mapped to known intergenic region. There were more highly expressed intervals with exonic expression than intronic or intergenic ones. B. Venn diagram illustration of the above result. Only intervals containing more than 100 reads were considered, as indicated by the dotted line in A.

After further dividing the genome into 10 kb intervals, most of the 10 kb intervals had no detected transcription (Figure 2A). Most of the reads in highly expressed intervals (> 1,000 RNA-seq reads) were mapped to known exons. Among intervals with 1,000 to 10,000 detected reads, more intervals had intergenic transcripts than intronic transcripts, with very few intervals having all three types (exonic, intronic and intergenic) of transcripts. For intervals with 100 or more RNA-seq reads, there were 3 times more intervals expressing intronic signal in E18 stage than in P7 stage. The E18 stage also had slightly more intervals with intergenic transcripts than the P7 stage, although the numbers of intervals with exonic transcripts were similar between the two stages (Figure 2B).

**Figure 3:**
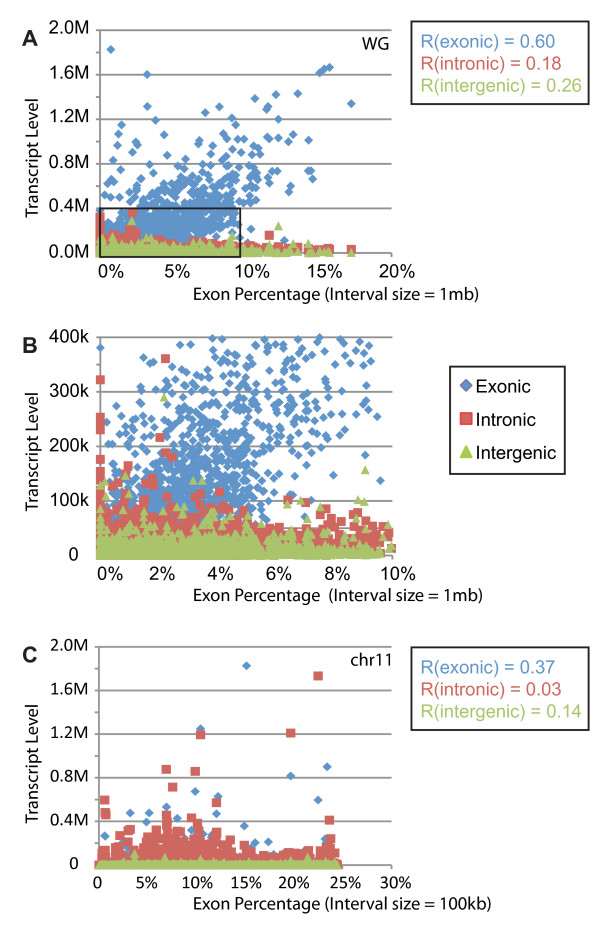
**Correlation between exon density (measured by percentage of exon regions) and expression level**. A. Interval size = 1 Mb. Regional percentage of exon vs. exonic expression (blue diamond), intronic expression (red square), intergenic expression (green triangle). B. Zoomed in view of the boxed region in A. C. Interval size = 100 kb. Only data from chromosome 11 were shown.

We found that expression level was positively correlated with the exon contents of a given interval. The higher exon percentage an interval had, the higher the number of detected RNA-seq reads in the interval. At 1 Mb interval size, the Pearson's Correlation Coefficient between exonic read number and exonic content percentage was 0.60; in contrast, the Pearson's Correlation Coefficient between intronic read number and exonic content percentage was only 0.18. The Pearson's Correlation Coefficient between intergenic read number and exonic content percentage was also quite low, at 0.26 (Figure 3A and 3B). At 100 kb interval size, the correlation decreased but the general trend was maintained (Figure 3C). While the exon-rich regions had more reads for exons than introns or intergenic regions, the exon-sparse regions had similar numbers of intronic and intergenic reads as the exon-rich region. In addition, at 1 Mb resolution, the exonic expression level had modestly positive correlation with both intronic (*R *= 0.43) and intergenic expression level (*R *= 0.37) in the same region (Additional file 1, Fig. S2 **A **and **B**). However, at 100 kb resolution, the aforementioned correlation became very weak (Additional file 1, Fig. S2 **C **and **D**).

### Transcriptomic comparison between neural tissues and other tissues

To understand the neural transcriptome characteristics, we compared mouse cortical RNA-seq data at E18 and P7 stages with available adult mouse brain, liver, and muscle RNA-seq data [18], as well as RNA-seq data from human embryonic stem cells (hESC) and neural cells (N1, N2, N3) immediately differentiated from hESC [7]. We first analyzed transcriptome properties at the chromosome level, using a method slightly modified from Mortazavi *et al. *[18] as detailed in the Methods section, and labeled as RPKM* (similar to RPKM; formula (1)).

In addition to the above-mentioned mapping of the 5 mouse datasets (E18 and P7 cortices, and adult brain, liver and muscle), we also mapped the 4 human RNA-seq datasets (hESC, N1, N2 and N3) onto the mouse reference genome (mm9 in the UCSC database; [21]). Based on the 85% identity calculated from coding regions between mouse and human genome previously [21], there would be on average 5 mismatches per 35 bp RNA-seq read length. We found that the threshold of 2 mismatches per 35 bp read achieved the best balance between specificity and sensitivity for this cross-species mapping. Increasing the number of allowed mismatches resulted in fewer uniquely and correctly mapped reads, while decreasing this number resulted in fewer total mappable reads. With the threshold of allowing maximum 2 mismatches for RNA-seq mapping, this would mean little cross-species mappable reads if the differences between coding regions of human and mouse were distributed evenly. Surprisingly, on average 6% of the total human RNA-seq reads could be mapped to the mouse genome, or 11.5% of the reads mappable to the human genome. The majority (80%) of the reads mapped to the mouse genome were also mapped to the human genome.

**Figure 4:**
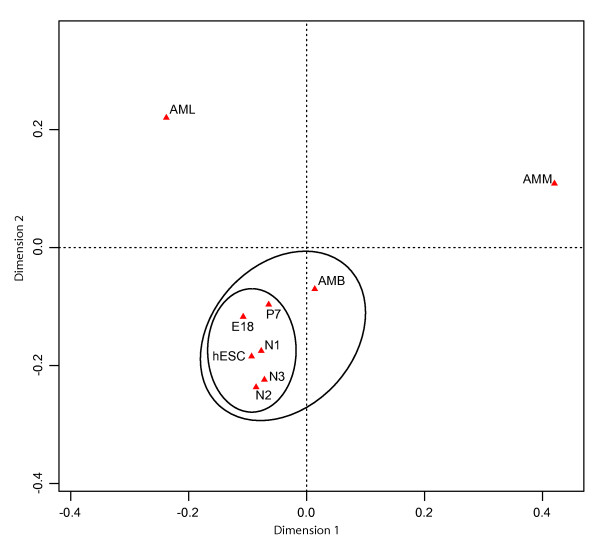
**Correspondence Analysis result on stage similarity based on comparison between chromosomal expression profiles**. Similar stages were closer to each other than dissimilar ones. Analysis done using ca package in R [22].

We then compared the chromosomal expression profile between mouse (E18, P7, AMB, AMM and AML) and human (hESC, N1, N2 and N3) samples. Despite the fact that smaller fractions of the human reads were mapped onto the mouse reference genome, largely due to DNA sequence similarity between these two species, both mouse and human neural data sets were highly similar in terms of expression level relative to total mapped reads at the scale of individual chromosomes (Additional file 1, Fig. S3A). Furthermore, all neural tissues had similar profiles to that of stem cells, but quite different from those of adult liver and muscle, particularly for some chromosomes, such as 5 and 7 (Additional file 1, Fig. S3B). To summarize the above information, we applied Correspondence Analysis on the chromosomal expression profile. The chromosomal expression distribution among all stages, using mouse genome as reference, was first measured in read counts (Additional file 2, Table S1). Correspondence Analysis (CA) was performed using the ca package in R [22]. The first two dimensions resulted from the CA could explain 96% of the differences in the original 9 × 21 dimensions, which indicated that the first two dimensions were representative (Additional file 2, Table S2). The result was plotted using these two dimensions (Figure 4). E18 and P7 were clustered together with hESC, N1, N2 and N3. AMB was also very close to the aforementioned cluster. AML and AMM were significantly further away than AMB.

As an alternative way to compare the mouse and human data, we also mapped the human data onto the human reference genome (hg19; [23]), for comparative analysis using previously identified syntenic/orthologous genomic regions between mouse and human [21,23]. These studies defined 217 conserved syntenic blocks between the human and mouse genomes. Chromosomal expression profiles in early-differentiated human neural cells were very similar to that of human embryonic stem cells (Additional file 1, Fig. S3C). Even with different chromosome numbers and organizations, neural chromosomal expression profiles were also very similar between human neural cells and mouse neural tissue samples between syntenic/orthologous genomic regions (Additional file 1, Fig. S3B, C). For example, the most highly expressed chromosome in mouse was chromosome 11, whose human counterpart is chromosome 17, which was the second most highly expressed chromosome in human. The most highly expressed chromosome in human was chromosome 19, whose mouse counterparts are distributed on chromosomes 7, 8 and 19, among which chromosomes 7 and 19 were also highly expressed in neural tissues.

To assess the variation in expression levels between chromosomes for different tissues/organs, we calculated the standard deviation for the distribution of individual chromosome expression level, or RPKM* values, for each mapped transcriptome dataset (Additional file 1, Fig. S3D). We found that the standard deviation for E18 was the lowest among mouse samples, while the standard deviation for the stem cells was the lowest in human samples (Additional file 1, Fig. S3D). These results indicated that the mouse E18 brain cortex and human embryonic stem cells use chromosomes more evenly than other organs/tissues.

It is well known that the brain has a very high metabolic rate, consuming a significant amount of energy while lacking substantial energy reserve tissues. Thus normal brain functions depend on mitochondria as the crucial energy provider. To examine the mitochondrial genome expression-level changes across different developmental stages, we plotted the normalized mitochondrial expression level, measured in RPKM*, across all nine datasets and normalized against the dataset size. We found that in human datasets, compared with stem cells, differentiated neural cells had a higher level of mitochondrial expression (Additional file 1, Fig. S3E), increasing from the N1 to N2 stages, then maintaining a similarly high level at the N3 stage. Similarly in the mouse brain, mitochondrial expression progressively increased from the E18 embryonic stage to the P7 neonatal stage, then to the adult stage. The adult mouse brain had a similar level of mitochondrial expression to that of the adult liver, while the adult mouse muscle had the highest level of mitochondrial expression among all analyzed organs/tissues, consistent with high energy demand for muscle contraction.

**Figure 5:**
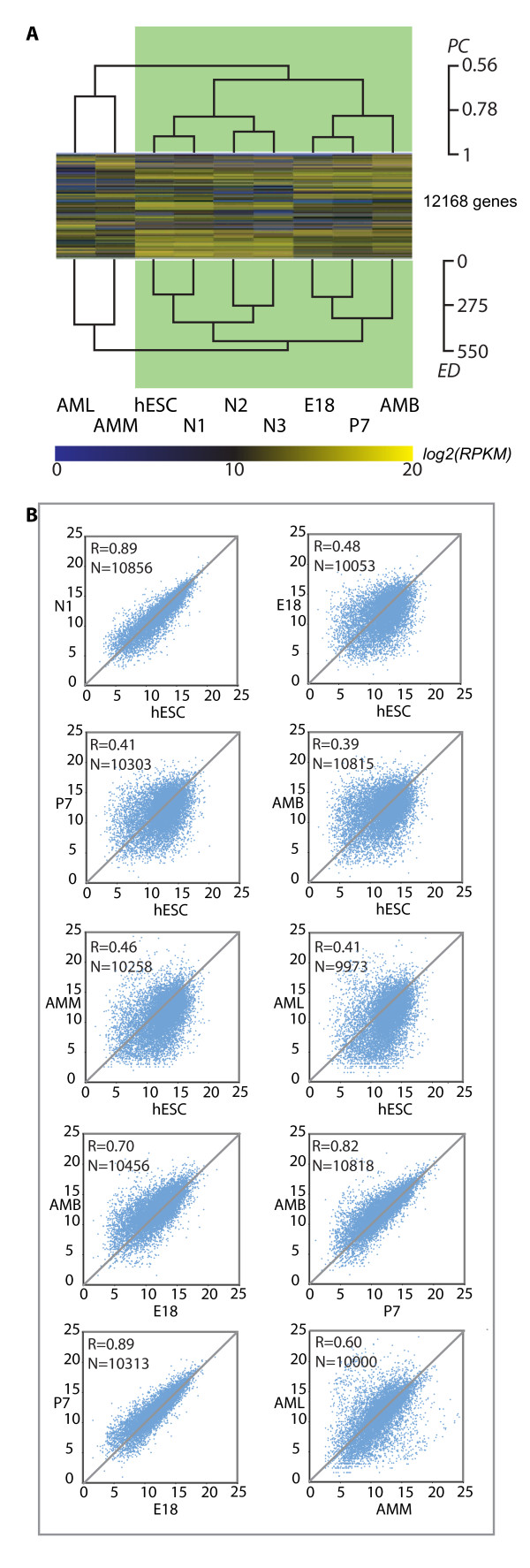
**Comparisons of human and mouse expression data**. **A**. Clustering of nine RNA-seq datasets using orthologous genes between human and mouse. Center panel: expression level heat map overview of 12168 orthologous genes. Expression level is measured in RPKM [18]. The calculated number is increased by 1 to ensure valid logarithm transformation, and then log_2 _transformed. The sample tree on top is derived from Pearson's Correlation (*PC*) distance matrix, while the one at the bottom is derived from Euclidean Distance (*ED*) matrix. Both trees support the same topology, which grouped all neural stages with stem cell stage, while left muscle and liver in a separate group. Scale of color representation of the expression level is at the bottom of the figure. **B**. Scatter plot of orthologous gene expression level between selected stages. Genes without detectable expression were not included.

To assess the similarity between neural and stem cell transcriptomes further, we compared the transcriptomes between human and mouse using only 1-to-1 orthologous gene pairs between these species. A total of 12168 orthologous gene pairs were identified using the MGI (http://www.informatics.jax.org) orthology database. The expression level of each gene was measured in RPKM, with the modification that detected base pairs from exons were used instead of read number in the RPKM formula to accommodate read length differences between datasets. Again the expression level for a given gene at a given stage was normalized against the RNA-seq dataset size. We then added 1 to the calculated expression level value for each gene, to ensure valid logarithm transformation. The calculated value was then log_2 _transformed. We first calculated the gene expression correlation between hESC and the rest of the samples. As expected, the correlation was high between hESC and the cells derived from hESC. Among the rest, E18 had the highest correlation with hESC (R = 0.61), and P7 had the second highest (R = 0.57). The data were then analyzed in MeV (MultiExpriment Viewer) from the TM4 suite [24]. After using different clustering methods (Hierarchical clustering and K-means) and different distance calculation methods (Pearson's Correlation and Euclidean Distance), we found that neural datasets from human and mouse were consistently grouped together with the stem cell dataset, separate from the liver and muscle datasets (Figure 5A). This suggested that the neural and stem cell transcriptomes were globally more similar in terms of orthologous gene expression than they were to liver and muscle transcriptomes.

We then further analyzed the correlation of the expression in two different tissues/stages among co-expressed genes between the tissues/stages (Figure 5B and Additional file 1, Fig. S6). Among all mouse samples, although E18 was the one with the highest correlation with hESC, E18 was still more similar to mouse neural transcriptomes in terms of expression level correlation. In particular, E18 and P7 transcriptomes were much more correlated with each other than with hESC, suggesting that the similarity between E18 or P7 cortex and hESC is relatively limited.

**Figure 6:**
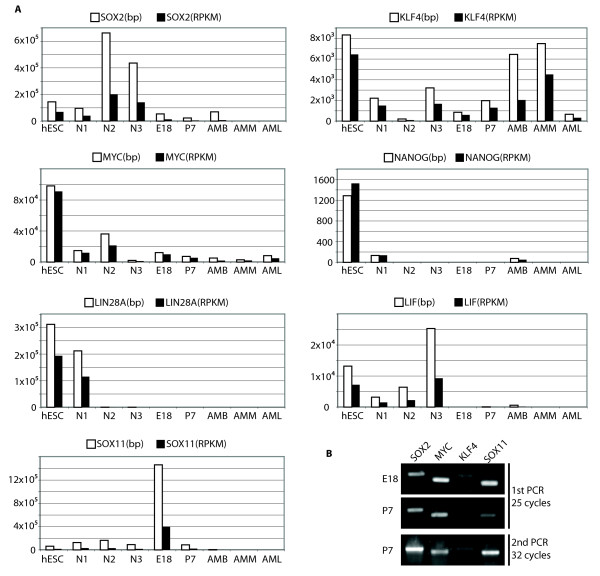
**RNA-seq levels and RT-PCR analyses of pluripotency-related genes**. A. Expression level of pluripotency-related genes across stages. Expression level was measured in both raw RNA-seq data size (bp) and RPKM methods. B. RT-PCR results showing the expression of *Sox2*, *Myc *and *Klf4*, with *Sox11 *as the positive control.

We also analyzed the genes associated with the pluripotency of stem cells. *Sox2*, *Myc*, *Oct4 *and *Klf4*, which are four genes that have been found in multiple studies to convert human and mouse somatic cells to induced pluripotent stem (iPS) cells [25-29]. Similarly, *Oct4 *and *Sox2*, plus two other factors, *Nanog *and *LIN28A*, were also able to induce iPS cells from human somatic cells [30]. We found that *Sox2*, *Myc *and *Klf4 *were detectable in all neural samples we analyzed (Figure 5A). Specifically, the E18 stage had the highest *Sox2 *and *Myc *expression among all mouse samples. However, *Nanog *and *LIN28A *were not detected at either the E18 or P7 stage. RT-PCR experiment was also carried out with primers specifically targeting *Sox2*, *Myc *and *Klf4*. The results supported the expression of *Sox2*, *Myc *and *Klf4 *in E18 and P7 cortices (Figure 6B). Due to the presence of multiple homologues of *Oct4 *(also called *Pou5f1*) in the mouse genome, neither RNA-seq nor RT-PCR could identify specific expression for *Oct4/Pou5f1*. Interestingly, *Sox11*, which encodes a transcription factor and was previously reported to be expressed in glial cells [31], was highly expressed in the E18 cortex and significantly down-regulated in the P7 cortex (Figure 6A &6B). Because cortical neurons are mainly generated at the late embryonic stage whereas glial cells are mainly generated in the postnatal stage, the high level expression of *Sox11 *in the E18 cortex suggests an additional role during early brain development besides its proposed function in glial cells.

### Expression characteristics of genes associated with neurodevelopmental disorders

**Figure 7:**
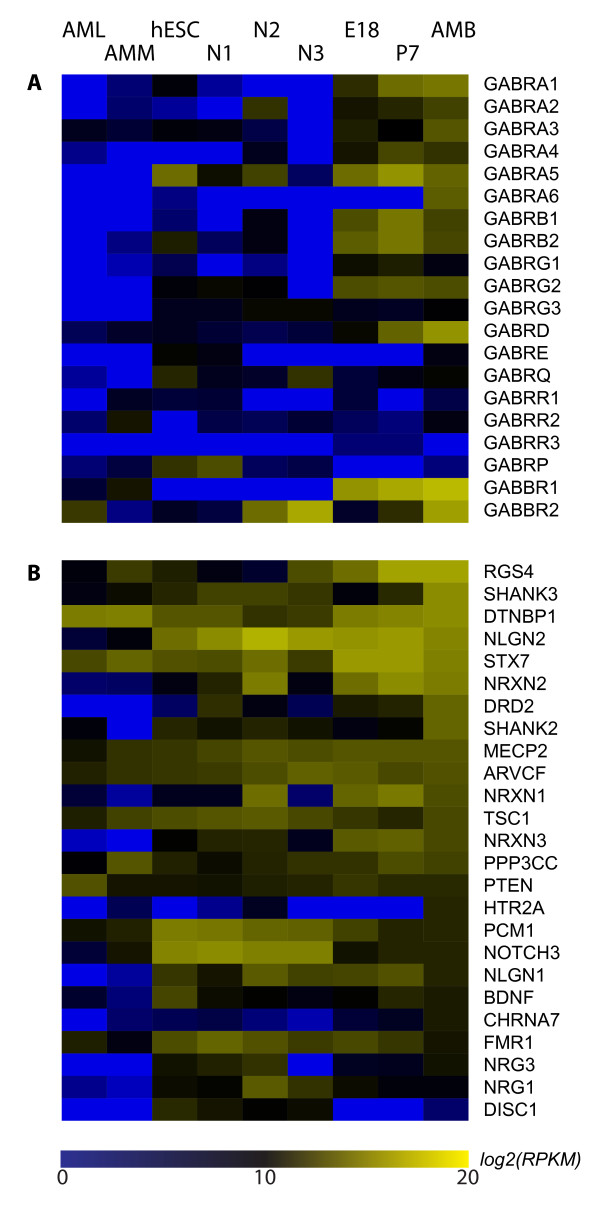
**Expression of genes associated with neurodevelopmental disorders**. A. Expression of selected GABAA receptor genes. B. Expression of selected Autism spectrum disorders associated genes.

We further analyzed the expression of genes potentially associated with neurodevelopmental disorders in both neural tissues and stem cells. Autism spectrum disorders (ASD), together with schizophrenia and mental retardation, are typically characterized as neurodevelopmental disorders. Genome-wide association studies (GWAS) have identified many genes related to ASD. Among these genes, many have been found to relate to the GABAergic neurotransmission system. Here, we analyzed the expression of 20 genes encoding different GABAA receptor subunits and 25 genes that have been proposed to be associated with ASD [32,33] (Figure 7A and 7B). Although GABA receptor genes in general showed low expression levels in non-neural tissue and stem cells, the gene for GABA_A_R alpha5 (GABRA5) subunit showed a very high level of expression in hESCs, which lacks a GABAergic system, suggesting a novel function of this gene in embryonic stem cells. Its expression was not detectable after initial differentiation (N1) and then observed again after further differentiating into neural cells (N2). It also showed an increase from E18 to P7, but a reduction from P7 to adult brain, consistent with more restricted localization in the adult brain [34]. In addition, GABRQ and GABRP also showed modest expression in hESCs. Furthermore, the gene encoding the GABA_A_R delta (GABRD) subunit showed the highest expression level among all GABA_A_R subunit genes in the adult brain. Since delta is specifically localized at extrasynaptic sites and mediates tonic inhibition rather than normal fast inhibition, this result emphasizes the importance of tonic inhibition in regulating adult brain activity. Finally, genes for GABA_B _receptor (GABBR1 and GABBR2) subunits showed significant differential expression during brain development, with GABBR1 dominant from E18 through adult brain while GABBR2 only expressed highly in the adult brain.

Among genes associated with ASD, *RGS4*, *DTNBP1*, *NLGN2*, *STX7*, *MECP2*, *ARVCF*, and *PPP3CC *all showed high-level expression from the embryonic to adult brain. One important finding was that while both *NLGN1 *and *NLGN2 *showed high-level expression at the E18 to P7 stages, consisting with their synaptogenic functions, *NLGN1 *expression was significantly reduced in the adult brain, suggesting that the relevant function might be fulfilled by other cell adhesion molecules. This is also consistent with the current understanding that many cell adhesion molecules can trigger glutamatergic synapse formation as NLGN1 does, but only NLGN2 is capable of inducing GABAergic synaptogenesis [35,36]. One surprising finding is that *DISC1*, a well-studied gene associated with schizophrenia [37], showed very low expression at the E18/P7 stages and still low in adult brain. However, *DISC1 *was modestly expressed in hESCs and the expression decreased after neural differentiation, suggesting that *DISC1 *might play an important role in stem cell functions.

### Detection of unusually high levels of unannotated transcript expression level at E18 and P7 stages

**Figure 8:**
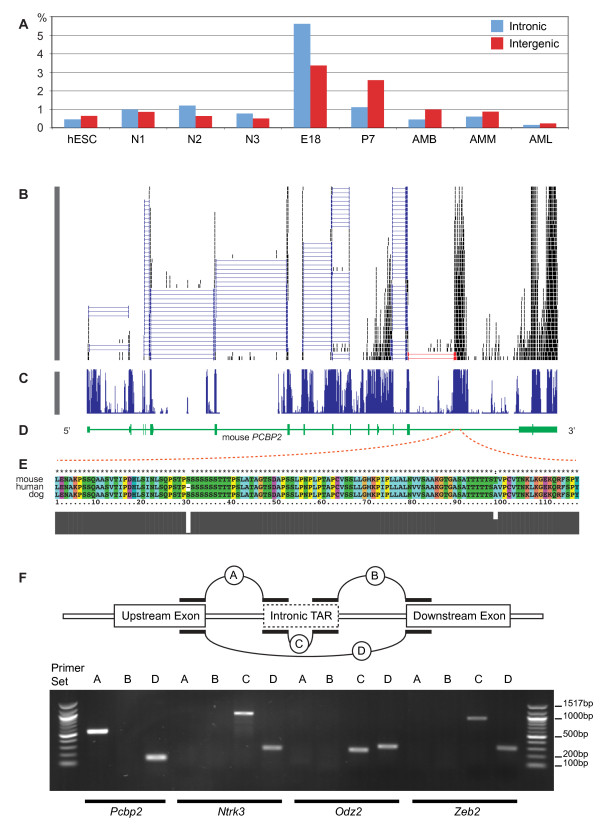
**Detection of unannotated transcriptionally active regions (TARs)**. A. Percentage of TAR data of each dataset. Column in red color represents intronic TAR, and column in blue color represents intergenic TAR. X-axis represents different datasets, while Y-axis represents the percentage. B to E: An intronic TAR found located inside mouse *PCBP2*. B: RNA-seq reads mapped by TopHat to this location. Each black bar represents a RNA-seq read. Because the coverage on the 3' end (right side) of the gene is too high, only part of the reads mapped at this region is shown. Each blue horizontal line connecting two bars indicates a splice-junction-spanning read (read split by splicing activity). Red lines highlighted such reads spanning the intronic TAR and the exon on its left. C: conservation score from 30-Way multi-species genome alignment. Notice the region with high conservation score co-localized with the intronic TAR. D: current *PCBP2 *gene model, with green box representing known exons. E: amino acid alignment of the ORF of intronic TAR and its human and dog homologues. Gray column plot below: each column represents the similarity of the amino acids in that column. F: RT-PCR validation for intronic TARs. For each intronic TAR inferred from RNA-seq mapping result, primers were picked to validate the connection between the intronic TAR and the flanking exon (upstream/downstream), between the flanking exons, as well as the expression of the intronic TAR.

To obtain an accurate set of unannotated TARs, we first subtracted the TARs overlapping with annotated exons, tRNAs or rRNAs. We then removed TARs overlapping with repeats. We also excluded TARs within 2 kb vicinity of the first and last exon to avoid promoter, TSS and TTS associated transcription activity, which have been previously studied [38-40]. We call the set of unannotated TARs generated from the aforementioned procedure the filtered unannotated TARs, and they include both intronic TARs and intergenic TARs. We found that the E18 stage had the highest percentages of both intronic and intergenic reads, at 5% and 3% of the E18 total data, respectively (Figure 8A). P7 stage had the second highest percentages, with about 1.1% and 2.6% for introns and intergenic regions, respectively. In comparison, almost all the other datasets had less than 1% of the data matching intronic or intergenic regions. Among human samples, surprisingly, the stem cell stage had the lowest percentage of unannotated TARs, while the neural progenitor cell N2 stage had the highest. This result is different from a previous report [7], which did not use methods that rigorously removed repeats as in this study.

### Concordant changes in expression levels between intronic TARs and flanking exons

To investigate the relationship between an intronic TAR and its flanking exons, we examined their respective expression levels (as measured in RPKM; [18]). There were 488 genes with intronic TARs at either the E18 or P7 stage. We found a strong positive correlation between the intronic expression and the flanking exonic expression. From E18 to P7, we found 436 genes with decreased intronic expression levels; among these 436 genes, 242 (56%) also had decreased exonic expression (Table 2). Even more strikingly, 52 genes had increased intronic expression level from E18 to P7 (Table 2), with 43 (83%) having increased exonic expression levels. The correlation was highly significant, with a p-value of 0.0001 from Fisher's Exact Test for the association of the exonic and intronic expression levels.

**Table 2:** Intronic and exonic expression level changes for genes with intronic TARs from E18 to P7.

	Also With Increased Intronic Expression	Also With Decreased Intronic Expression
Number of Genes With Increased Exonic Expression	43	194

Number of Genes With Decreased Exonic Expression	9	242

### Few unannotated TARs were connected with known exons

The strong concordant correlation between the previously unannotated intronic TARs and flanking exons suggested that the intronic TAR and its flanking exon might be parts of the same RNA transcript. To test this hypothesis, we focused on the E18 and P7 datasets, which had the largest percentage of filtered unannotated TARs. A paired-end read with one end located in the unannotated TAR and the other in a known exon would be strong evidence that this intronic TAR and the known exon are parts of the same mRNA. However, it is in principle possible that the mapping positions could be erroneous. In addition, the existing mathematical and statistical models for determining the connection between TARs [8] are designed for RNA-seq data from cDNAs generated with random primers. They are not applicable to poly-dT primed data, which have a 3' bias. So we first devised a model suitable for both priming techniques (Methods; formula (2)), which reports the presence of the physical connection between expressed TARs and known exons. Using known adjacent exons and single exon genes (SGEs) with detected reads as positive and negative controls in a simulation, the formula had success rates of 93% and 100%, respectively.

Using formula 2, we found that only a very small percentage of the unannotated intronic TARs were connected with known exons (Table 3) as supported by the RNA-seq reads. Although a large fraction (70% for E18, 60% for P7) of the unannotated TARs was found to be connected to other regions mapped elsewhere, these mapped regions were very short, fragmented and unannotated regions with low read coverage. Surprisingly, some unannotated TARs were connected to read ends that mapped to multiple chromosomes, possibly due to mapping to repetitive sequences, erroneous mapping or possible cross-chromosome splicing (*trans*-splicing), a rare phenomenon that was previously observed [41].

**Table 3:** Physical connection between unannotated TARs and known exons.

		E18 Brain Cortex		P7 Brain Cortex	
		
		Intronic TAR	Intergenic TAR	Intronic TAR	Intergenic TAR
**Connected with Known Exons**		19	2	13	2

**Not Connected with Known Exons**	**Standalone**	117	45	28	41
	
	**Non-Standalone (**Same Chromosome**)**	404	71	123	46
	
	**Other (**Non-Standalone Multi-Chromosome**)**	14	8	4	5

**Total**		**554**	**126**	**168**	**94**

### Comparing the intronic TARs with known mRNA and EST in NCBI databases

To test whether there is other evidence for the intronic TARs, we searched the data in NCBI's cDNA/mRNA and EST databases. Among 554 intronic TARs detected at E18 stage, 176 (32%) had no matches in NCBI databases. Similarly, among 168 intronic TARs at P7 stage, 49 (29%) had no matches in NCBI databases. Therefore, our results provide the first evidence for these TARs being expressed. Among the matching NCBI database entries, 11 (2%) of the 378 for the E18 stage and 7 (4%) of the 119 for the P7 stage were from the same stages, but none of them was from the brain cortex.

We then examined the splicing pattern of the mRNA and EST records matched to our detected intronic TARs and found two classes of intronic TARs: (1) with records suggesting that the TARs were standalone, without connection to known exons; (2) with some records suggesting that the TARs were standalone while other records suggesting that they were connected to known exons. 304 out of 378 (80%) intronic TARs at E18 and 75 out of 119 (63%) intronic TARs at P7 belonged to the first class. For the second class of intronic TARs, on average, the ratios for records supporting standalone transcripts to those for connections to known exons were 4.2 and 2.8 for the E18 and P7 stages, respectively. Taking together, the comparison with NCBI's cDNA/mRNA and EST databases strongly suggested that most of our detected intronic TARs were not connected with known exons and thus were novel transcripts.

### Comparing the intronic TARs with known records in miRbase and lncRNAdb

We then compared our intronic TARs in miRNA database miRbase [42] and long non-coding RNA database lncRNAdb [43]. Although we found no significant hits in these two databases for any intronic TARs observed at P7 stage, we did find 12 and 6 hits for intronic TARs at E18 stage in miRbase and lncRNAdb, respectively (Additional file 2, Table S5 and S6). However, all 6 intronic TARs with hits in lncRNAdb were mapped to the same lncRNA, B2 SINE RNA, which was from a SINE repeat element. In addition, 11 of the 12 intronic TARs having hits in miRbase mapped to the same miR-1935 miRNA, and the remaining one mapped to miR-153-2. Otherwise, we did not detect significant hits for other types of RNAs.

### Sequence conservation and coding potential of intronic TARs

To obtain clues about possible function of the intronic TARs using sequence similarity to other mammalian genes, we investigated whether unannotated TARs corresponded to any highly conserved region using the 30-Way Multiz Alignment & Conservation track in UCSC Genome Browser [44]. We found that there were 554 and 168 unannotated TARs at E18 and P7 stages, respectively; among these, 67 in E18 and 21 in P7 matched regions of highly conserved sequences. For example, a TAR on chromosome 15 (102324092-102324772) was localized to an intron of the mouse *PCBP2 *gene encoding the major cellular poly(rC)-binding protein [45]. In addition, there were RNA-seq reads spanning this intronic TAR and the upstream exon (Figure 8B, red reads), indicating that this previously unannoted TAR was spliced with a known exon. Moreover, this TAR had a significant overlap with a highly conserved region located in the 3' most intron, which was identified by mammalian conservation study using 30-Way Multiz Alignment & Conservation track data (Figure 8C and 8D) [44]. An Open Reading Frame (ORF) was also predicted inside this TAR and was conserved among the *PCBP2 *genes of human and dog (99% similar in amino acid sequences; Figure 7E), but not opossum. PCR and ABI 3730 resequencing results further verified that this TAR is indeed part of an mRNA (Figure 8F) with a connection between this TAR and the upstream exon, consistent with RNA-seq results. However, PCR product between this intronic TAR and the downstream exon was not detected, in agreement with the RNA-seq results. This TAR was very likely to represent an alternative 3' UTR with a potential coding region.

In addition, an intronic TAR with an ORF inside the *ATP2B1 *gene located on chromosome 10 (98481907-98482067) shares 99.3% identity to the 20^th ^exon of human *ATP2B1 *isoform a (*ATP2B1a*) (Additional file 1, Fig. S4). Human *ATP2B1 *has two splicing variants: *ATB2B1a *and *ATB2B1b*, which differ in the usage of the 20^th ^exon. Previous studies showed that *ATP2B1a *has a specific expression at synapses whereas *ATB2B1b *is expressed in most tissues [45,46]. Thus this TAR is likely to encode a neuron-specific exon of the mouse *ATP2B1 *gene. We also found another expressed region on chromosome 7 (112781296-112781396) that shares 87.5% identity with a part of the second 3' UTR exon of the human *Trim3 *gene (Additional file 1, Fig. S4). *Trim3 *(or *BERP*) is expressed in the brain and encodes a RING finger protein that regulates *GABAR *cell surface expression [47]. Another intronic TAR located in the *NRXN1 *gene on chromosome 17 (90854147-90854636) has the potential for coding Neurexin 1, a neuronal cell adhesion molecule interacting with neuroligins to promote synapse formation and maturation [48]. The ORFs in these intronic TARs were highly similar to parts of human *ATP2B1*, *BERP *and *NRXN1 *genes, respectively. A number of other intronic TARs, such as those in *CHD3*, *TSC22*, and *SRCAP*, were either similar to known human exons or supported by mouse gene predictions and mRNA and/or EST data in the NCBI database.

Three other intronic TARs were located, respectively, in the *Zeb2 *gene on chromosome 2 (44953049-44955802), the *Ntrk3 *gene on chromosome 7 (85484006-85485464), and the *Odz2 *gene on chromosome 11 (36491704-36492013), within introns that are more than 10 kb long. These TARs did not match mRNA or EST records in the NCBI database, nor were they similar to protein sequences in the NCBI database. Nevertheless, these three TARs were conserved in rat, human, dog and opossum genomes, matching annotated introns in the orthologous genes in human and rat (Additional file 1, Fig. S5). Our RNA-seq data did not detect physical connection between the TARs and known exons; the lack of connection between the TARs and the flanking exons were further supported by the observations that PCR was successful when both primers were located inside a particular intronic TAR, but not able to generate products when a primer in the intronic TAR region was combined with another primer in one of the flanking exons (Figure 8F). As a control, the correct PCR product was obtained using primers matching the two flanking exons of the given intronic TAR (Figure 8F). Therefore, these three intronic TARs were most likely standalone transcripts that were not linked with the flanking exons.

### Genes with intronic TARs were over-represented in GO terms closely associated with neural development

**Figure 9:**
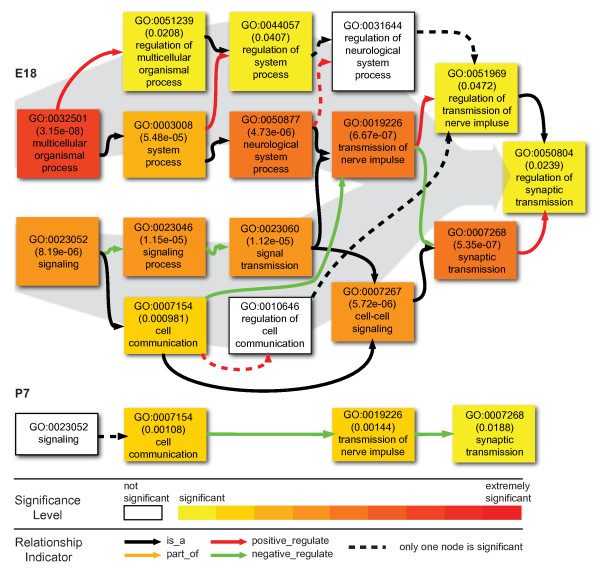
**Neural signaling GO terms significantly overrepresented by genes with intronic TARs**. The coloring of the node box indicates the statistical support for the overrepresentation. The color scale at the bottom of the figure illustrates the different levels of statistical support. The node box with white color means that that node was not statistically overrepresented. However these are included to illustrate the relationship between statistically significant nodes.

**Figure 10:**
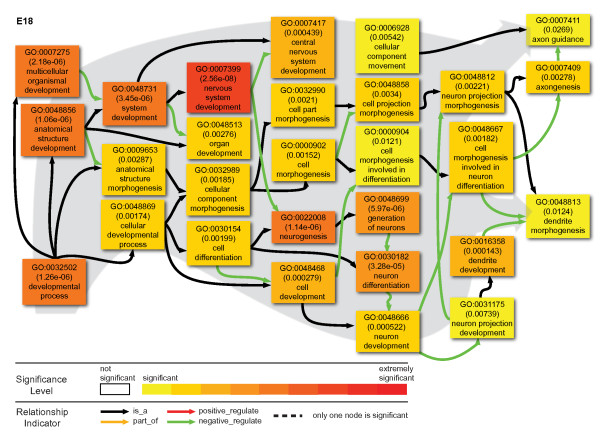
**Neurogenesis GO terms significantly overrepresented by genes with intronic TARs**. The coloring of the node box indicates the statistical support for the overrepresentation. The color scale at the bottom of the figure illustrates the different levels of statistical support. The node box with white color means that that node was not statistically overrepresented. However these are included to illustrate the relationship between statistically significant nodes.

**Figure 11:**
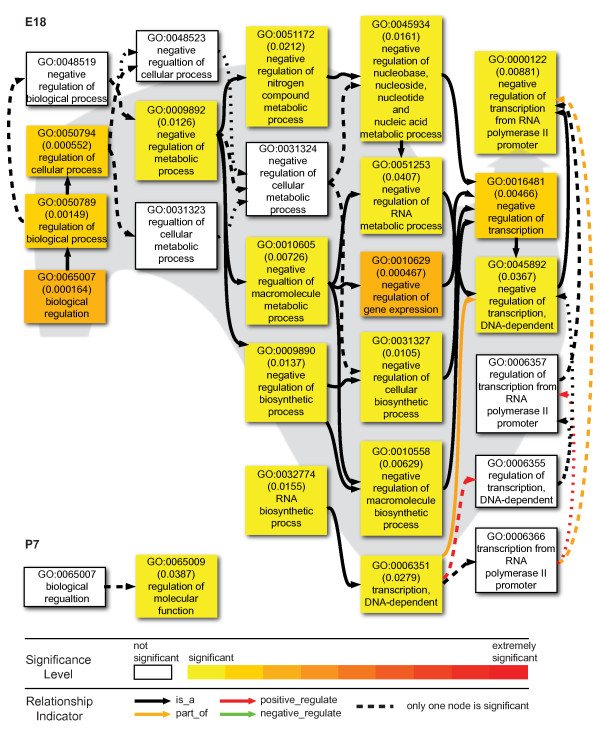
**Regulation GO terms significantly overrepresented by genes with intronic TARs**. The coloring of the node box indicates the statistical support for the overrepresentation. The color scale at the bottom of the figure illustrates the different levels of statistical support. The node box with white color means that that node was not statistically overrepresented. However these are included to illustrate the relationship between statistically significant nodes.

Although we have found that few of these intronic TARs were physically connected with the exons of the corresponding genes in the same mRNA, the fact the same genomic regions encode both the transcripts with exons and the intronic TARs suggests some association between these intronic TARs and the exonic genes. To further examine the functional implication of the intronic TARs, it is informative to study the corresponding genes. To study the nature of proteins encoded by the genes with intronic TARs, we analyzed their enrichment in Biological Process Gene Ontology (GO) using agriGO [49]. The GO annotations for all expressed genes at either stage were used as a reference for comparison to determine possible enrichment of specific GO categories. A total of 316 unique genes contained filtered intronic TARs among a total of 10657 genes with detected reads at E18, while only 119 genes contained filtered intronic TARs among 10901 genes expressed at P7. E18 data had 59 statistically overrepresented GO terms, but P7 data only had 4 statistically overrepresented GO terms, 3 of which are shared between these stages. The 60 overrepresented GO terms could be mapped onto only 3 major branches: neural signaling (Figure 9), neurogenesis (Figure 10) and regulation (Figure 11). The GO terms of these branches are closely related with neural developmental events occurring at E18 stages.

For neural signaling related GO terms at E18, two subgroups form largely parallel interactions: the first subgroup mainly functions in regulating neural system process, while the second subgroup carries out signal transmission (Figure 9). The terminal node was the regulation of synaptic transmission, which combines the aforementioned two functions. Among the nodes with strong statistical support were "transmission of nerve impulse", "neurological system process", and "synaptic transmission". Another interesting aspect is that the regulatory relations in the first subgroup were positive, while they were negative in the second subgroup. For neural signaling related GO terms at P7, however, only one function group, similar to the aforementioned 2^nd ^subgroup, was identified. It had 3 nodes and the regulatory relations between the nodes were negative (Figure 9).

Nearly half (28, all at E18) of the enriched GO terms were for neurogenesis, with extensive interconnections between nodes and no obvious functional subgroups (Figure 10). The functions of these nodes include many aspects of neural development, such as cell morphogenesis, neurogenesis and neuron differentiation, eventually diverging into two termini: (1) axongenesis and axon guidance and (2) dendrite morphogenesis. It is also striking that all regulatory relationships were negative.

As shown in Figure 10, the third major group of overrepresented GO terms at E18 was for regulation. They were mainly about negative regulations, consistent with the negative regulatory relations identified between the majority of the nodes for neural signaling and neurogenesis groups. Specifically, strong statistical support was found for negative regulation of "metabolic process", of "gene expression" and of "biosynthetic process", ending in that of RNA polymerase II-dependent transcription. For P7, only one node received strong statistical support: regulation of molecular function (Figure 11).

Because axon guidance is critical for correct formation of the neural circuit during neural development, we then further analyzed the axon guidance node at E18. A total of 10 genes with filtered intronic TARs were assigned to this node by GO. According to the KEGG Pathway database [50], among the 10 genes, *Robo1*, *Robo2*, *Nrp1*, *Dcc*, *Ephb1 *and *Ephb2 *were all receptors involved in axon guidance pathway. They were all involved in the regulation of the cytoskeleton dynamics and axon repulsion activity. The average ratio of intronic read number to exonic read number for these 10 genes was 3.9 at E18, but was 1.8 at P7 (Table 4). As a comparison, we also examined three well-known genes: *Myc*, β-actin (*Actb*) and tubulin (*Tuba1a*), and found that their expression levels (exonic reads) were very similar between E18 and P7. However, the average ratios of intronic read number to exonic read number for these three genes were 0.023 and 0.051 at E18 and P7, respectively. These ratios were less than 1/100 (E18) or 1/30 (P7) of those for the 10 axon guidance associated genes. From a different perspective, for the 10 axon guidance associated genes, the average exonic reads ratio (E18/P7) was 1.61, suggesting a slight reduction in expression. In contrast, while the average intronic reads ratio (E18/P7) was 3.67, representing a much bigger reduction of the intronic transcripts. As a reference, the 3 house keeping genes had an average ratio of 1.09 for E18/P7 exonic reads, whereas the intronic read numbers for the 3 genes were too low to compare accurately. Therefore, the 10 axon guidance-related genes had significantly more intronic reads than exonic reads (P-value = 0.001, Chi-square with Yates correction) and more so at E18 than P7 (P-value = 0.001), suggesting a possible role of these intronic transcripts in modulating axon guidance at E18 cortex. **Conclusions**

**Table 4:** Exonic and intronic RNA-seq read number comparison between E18 and P7 for exon guidance related genes.

Gene Name	Number of detected reads in E18		Number of detected reads in P7	
	
	From Exon	From Intron	From Exon	From Intron
Slit3	43	1012	96	827

Robo2	2123	4900	1415	796

Robo1	1569	1872	425	362

Nrp1	991	1688	533	416

Nrcam	775	1150	1434	730

Klf7	1032	826	1088	328

Gli3	295	133	83	51

Ephb2	697	845	548	456

Ephb1	2006	1773	834	435

Dcc	1294	6509	281	1246

**Read Ratio (Intron/Exon)**	**3.860**		**1.802**	

**Intronic Read Ratio (E18/P7)**	**3.67**			

**Exonic Read Ratio** **(E18/P7)**	**1.61**			

Myc	358	13	229	8

Actb	8847	144	8236	503

Tuba1a	4029	63	3724	214

**Read Ratio (Intron/Exon)**	**0.023**		**0.051**	

**Intronic Read Ratio (E18/P7)**	**0.30**			

**Exonic Read Ratio** **(E18/P7)**	**1.09**			

### Neural and stem cell transcriptome

In this study, we have investigated global characteristics of embryonic and neonatal neural transcriptomes, and compared with transcriptomes of the adult brain and embryonic stem cells. We found that embryonic and neonatal brain cortex transcriptomes correspond to most genomic regions at large scale of megabase intervals, but are unevenly distributed with positive correlation to exon density. In addition, neural transcriptomes are similar to that of embryonic stem cells, more than those of liver and muscle, in several features including chromosome level expression (Additional file 1, Fig. S3A and B), and expression pattern of orthologous genes (Figure 5). Also, the E18 brain cortex transcriptome and hESC transcriptome showed relatively even chromosomal distribution and had lower mitochondrial expression.

Other than these global similarities, we noted another shared characteristic between neural expressed genes and genes important for pluripotent stem cells. Specifically, three genes, *Sox2*, *Myc *and *Klf4 *were detectable in all six neural samples (Figure 6), with high levels in E18 or P7 transcriptomes. The expression of these genes suggests that neural cells might need fewer factors to be converted to stem cells. Indeed Kim *et al *found that only two factors (*Oct4 *with either *Klf4 *or c-*Myc*), instead of four, were needed to revert neural stem cell to iPS cells [51]. Therefore, the similarity in transcriptome, including the expression of specific genes, such as *Sox2*, *Myc *and *Klf4*, between neural cells and stem cells suggests that neural cells might retain certain stem cell properties and have greater potential to be reprogrammed to be pluripotent.

### Intronic TARs as standalone RNA regulators in early brain development

The mapping results of our transcriptome datasets revealed significant levels of intronic reads. We found that only a small portion of the intronic transcripts that we detected was on the same RNAs with any known exons. Recently, Klevebring *et al. *have reported that about 50% of the intronic expression was actually from the antisense strand [11], different from the sense exon-containing mRNAs of the same gene. Thus the intronic TARs detected here share some characteristic with the antisense transcripts, although our data lacked the strand information. Our finding that the level of intronic transcript is positively correlated with that of flanking exons is consistent with previous studies that antisense transcription may have both concordant and discordant regulation relative to the adjacent exons [3]. Furthermore, Faghihi *et al. *also reported regulation involving antisense RNA not mediated by the conventional RNA interference pathway [52], indicating additional mechanisms are important. Our results that the E18 brain cortex has significantly higher levels of intronic transcripts than other tissues/organs strongly suggest that such non-coding transcripts play important roles in regulating gene expression during embryonic brain development.

We also found that the mouse E18 embryonic brain had a concordant relation between intronic transcript and flanking exonic expression. This is unlike previous studies showing preferential localization of antisense transcripts in the upstream and downstream regions of the gene [53-55]. Our data have further indicated that the E18 embryonic brain showed enrichment of genes with intronic TARs in GO categories that are closely associated with neural functions. The E18 cortical neurons are actively engaged in neurogenesis, including axonogenesis and synaptogenesis. For the significant GO terms associated with neurogenesis, all the regulatory relations between nodes were negative (Figure 10). Moreover, an entire group of significant nodes was about negative regulation (Figure 11). However, at P7, intronic TARs were no longer associated with either neurogenesis or negative regulation. These findings suggest the involvement of intronic TARs in stage-specific regulation of neural developmental.

Recently, a subset of long ncRNAs was found to have an enhancer-like function [56]. Our data also indicated a correlation between the change in intronic transcript expression level and the change in the expression level of the corresponding gene. For example, the increased intronic expression is correlated with increased exonic expression for 10 axon guidance associated genes, whereas such correlation was not found for a control set of 3 housekeeping genes. The positive correlation in expression change between intronic TARs and the flanking exons further supports the idea that they have regulatory interactions, although it is formally possible that the intronic transcripts have functions unrelated to the genes represented by the flanking exons.

Our transcriptome analyses have revealed possible important mechanisms for gene function and its regulation in the developing brain, and uncovered a strong similarity to stem cells. These results provide a number of novel insights regarding neural developmental gene functions that can be further investigated using molecular genetic, biochemical and electrophysiological experiments.

## Methods

### RNA-seq mapping

RNA-seq data for hESC, N1, N2 and N3 were obtained from NCBI Sequence Read Archive SRP002079. RNA-seq data for adult mouse brain, liver and muscle tissues were obtained from NCBI Sequence Read Archive SRA001030. RNA-seq data for mouse embryonic day 18 and postnatal day 7 brain cortices were the same as described previously [9]. Its NCBI Sequence Read Archive accession number is SRP007262. The protocol for dissection of the mouse cortex was approved by IACUC committee of Pennsylvania State University and in accordance with the US Federal guidelines. All quality scores were then transformed into FASTQ ASCII code by original quality score plus 64. TopHat was selected for mapping these RNA-seq data. SRP002079 data were mapped onto human genome (UCSC hg19, NCBI Build 37), and the rest were mapped onto mouse genome (UCSC mm9, NCBI Build 37), both with the following parameters: --solexa-qual, -g 1. The same parameters were used when we mapped all nine datasets onto mouse reference genome. Although TopHat was instructed to report only unique hit (-g 1), it sometimes could not fully suppress multiple hits (personal communication with Cole Trapnell, TopHat author, on Feb 22^nd ^2011). Results were then further screened against RepeatMasker [57] database of the corresponding species to further eliminate possible ambiguous hits.

### Normalizing against data size and chromosome size

Normalization was done according to previously published RPKM method [18] with the following adjustment: length normalization was done against chromosome size *L_C _*when we were studying the chromosomal expression level distribution. Also, unlike the original RPKM concept, detected base pair size *C_bp _*was used instead of read numbers to accommodate different read-lengths from different RNA-seq datasets (33 bp, 35 bp and 36 bp). For the same reason, total mapped base pairs *N_bp _*was used in normalization against data size. And thus the RPKM* label was used here to distinguish these differences:

(1) $$RPKM^{*}=\frac{C_{bp}\times 10^{12}}{N_{bp}\times L_{C}}$$

Data size normalization was done against mappable data size instead of original data size generated from sequencer. This was to accommodate systematic sequence quality and mapping percentage differences from different datasets.

### Unannotated transcriptionally active region (TAR) calling

After the RNA-seq data were mapped to the target genome, regions with continuous read coverage that were within close proximity to each other were then chained together, thus forming the transcriptionally active regions (TARs). Only TARs longer than 100 bp and with more than 5X coverage were considered. These TARs were then compared with UCSC Known Gene [58]. TARs that did not overlap with UCSC Known Gene annotation features were then compared with known tRNA annotation [59] and custom-complied rRNA annotation. To further eliminate possible false-positives from repeat, TARs that were not included in any of the above annotations were then mapped back to genome with BLAST [60]. All regions with significant hits elsewhere in the genome were discarded. The remaining unannotated TARs were then filtered by their distance to their nearest exons. All unannotated TARs that were too close to known exons or genes were discarded as these may originated from previously reported small exon variations [61].

### RT-PCR validation for intronic TARs and the connection between intronic TARs and flanking exons

Sample preparation and RNA extraction were done according to the procedures described previously [9]. For the RT-PCR experiment, total RNA was isolated from mice E18 and P7 cortical tissues by using Ambion RNAqueous-Midi Total RNA Isolation Kit (Catalog#1911). One microgram of RNA was reverse transcribed into cDNA by using Biolabs DyNAmo cDNA Synthesis Kit (Catalog# F-470L).

To validate the expression of several pluripotency-related genes: approximately 1/20 of the first strand cDNAs was used as a template for PCR with gene-specific primers. PCR was carried out for 25 cycles of 94°C for 20s, 54°C for 30s, and 74°C for 40s. 10 ul of PCR products was separated on 0.8% (w/v) agarose gels containing ethidium bromide and visualized by UV light. A secondary PCR was performed for P7 with same primers by using 1 ul first round PCR products, for 32 cycles.

To validate the expression of specific intronic TARs, primer sequences were chosen within the intronic TAR, between the intronic TAR and the upstream exon, between the intronic TAR and the downstream exon, and between the upstream exon and the down stream exon. RT-PCR was carried out using the cDNAs as template with Taq polymerase for 22 cycles (add the temperature info). PCR product was sequenced at the Genomics Core Facility at Penn State using an ABI 3730 machine.

### Models to determine the physical connection of unannotated TARs with known exons/transcript for both poly-dT and random primed RNA-seq data

The general question can be abstracted to how to determine whether a given detected transciptionally active region (TAR) was on the same RNA with other exons/transcripts using paired-end information, i.e., there was a physical connection between the given TAR and another exon. If a given unannotated TAR is long and has many internal RNA-seq reads, its number of paired-end reads with one end located at a known exon should mean differently if a given unannotated TAR is short and with comparably less internal RNA-seq reads. We thus propose that the support for the aforementioned physical connection between the unannotated TAR and a known exon should be evaluated as a function of the length of the given unannotated TAR, the coverage (number of internal RNA-seq reads) of the unannoated TAR and the number of paried-end RNA-seq reads linking this unannotated TAR and another known exon. RNA-seq mappers also tend to have a lower mapping capability if they need to map a partial read at the end of an exon or TAR.

We first assumed that the paired-end read distribution inside a given TAR were either in a uniform distribution (in the case of using random primer), or in a skewed distribution (in the case of using ploy-T primer). Under this assumption, if the given TAR was part of a larger transcript, the number of paired-end reads at its end(s) should be similar to the average number of paired-end reads over the entire TAR. The following formula was used to calculate the estimated number of paired-end reads (*Ne*) at only one end of the given TAR, on the assumption that this TAR was part of a larger transcript:

(2) $$Ne=\frac{Ni\times Lc}{2\times Lr}-\frac{T\times Ni\times \left(Ls-M\right)}{Lr}$$

*Ni*, two times the total number of paired-end reads with both ends located inside the given region (to reflect each end in a pair).

*Ls*, read length for one end of a paired-end read.

*Lc*, clone length of a paired-end read, which is 2×*Ls *plus the insert size.

*Lr*, length of the given TAR.

*M*, maximum number of allowed mismatches of the mapping algorithm.

*T*, a correction factor. Splice junction spanning reads will have two partial matches to two discrete genomic regions. This value represents the success rate of the algorithm in mapping partial reads to the end of a given region, normally between 0 and 1. 0 means the algorithm cannot map partial reads to the end of a given region. 1 means the algorithm can map 100% of the partial reads to the end of a given region. Given the fact TopHat is designed to do RNA-seq mapping, the *T *value we picked was 0.99.

If data indicated that there was a significant amount of links from the given TAR to both upstream and downstream exons, the *Ne *should be doubled since there should be reads covering both ends of the given TAR. If the size of the given region *Lr *was smaller than that of the clone length *Lc*, then by theory all paired-end reads from this TAR should be reads linking this given TAR with other region(s).

If the actual number of paired-end reads connecting a given TAR with other regions was significantly less than (in this case we used 20%) *Ne*, the given TAR is thought to be a standalone transcript. Otherwise, this region was inferred as non-standalone, which means some level of splicing activity. More specifically, if a significant portion of the aforementioned paired-end reads had the other ends located in annotated exon(s), this given TAR was thought to be part of a known transcript. However, if the aforementioned reads were connecting more than one chromosome, then this TAR was thought to be multi-chromosome linked.

### Testing model effectiveness

To test the effectiveness of the proposed formula (formula (2)) in determining the connection between a given unannotated TAR and known exon(s), we must have positive controls that are known to be detectable in our dataset and are also known to be on the same RNAs with known exons. An expressed exon from a multi-exon gene would meet this requirement and should be able to serve as our positive control. To ensure that these exons were truly connected to known exons by RNA-seq reads, these exons were selected by hand through manual inspection of the RNA-seq mapping results using Integrative Genomics Viewer (IGV, http://www.broadinstitute.org/igv). For each selected exon, we made sure that there were multiple reads spanning the selected exon with at least one other known exon. To ensure a true representation of the genome wide situation from our test data, these selected exons were picked from different chromosomes, with different RNA-seq read coverage, different locations within a given gene and different relative distances to the 3' end (Additional file 2, Table S3). The proposed formula (formula (2)) was able to identify such exons as being physically connected with known exons with a success rate of 93%.

We also performed a negative control test to determine the effectiveness of the proposed formula in determining whether a given transcript has a physical connection with any known exon(s). Single exon genes (SEGs) in mouse genome were selected as the negative controls since they are known to be a standalone transcript. We first identified a list of SEGs which had RNA-seq reads in our dataset (Additional file 2, Table S4). The proposed formula was able to identify these selected SEGs as not being physically connected with any known exon(s) at a success rate of 100% (14% of the selected SEGs were determined, however, as multi-chromosome linked).

### Gene Ontology (GO) analysis

Reference mouse GO annotation was obtained from the Jackson Laboratory's MGI site (http://www.informatics.jax.org). Expressed genes were inferred from RNA-seq mapping results mapped to UCSC Known Gene. Expressed genes were then compared with reference mouse GO annotation. Identifier conversion between the UCSC Known Gene and the GO annotation was done using in-house script. Among all GO terms, only Biological Process GO terms were analyzed. We first calculated the number of genes mapped to a given GO term. For a gene with multiple GO terms, all terms were considered because one gene may be involved in multiple biological processes. If one GO term node was counted, all its parental nodes were excluded. Four sets of GO annotation were produced using the aforementioned procedure: all expressed genes in E18, all expressed genes in P7, genes with intronic TAR(s) in E18 and genes with intronic TAR(s) in P7.

For a given stage, GO annotation for the entire transcriptome and GO annotation for only genes containing intronic TARs were compared using agriGO server [49]. The statistical significance was determined by Fisher's Exact Test, with Bonferroni Correction. The p-value threshold was preset at 0.05 and only GO terms with more than 5 hits were reported.

## List of abbreviations

TAR: transcriptionally active region; hESC: human embryonic stem cell; N1: early initiation stage of hESC; N2: neural progenitor derived from hESC; N3: early glial-like cell derived from hESC; E18: embryonic day 18 brain cortex; P7: postnatal day 7 brain cortex; AMB: adult mouse brain; AMM: adult mouse muscle; AML: adult mouse liver; RPKM: reads per kilobase of exon(s) per million mapped reads; GO: gene ontology;

## Competing interests

The authors declare that they have no competing interests.

## Authors' contributions

HM designed research; YS and YH performed research; YS, YW and GC analyzed data; YS, YW, GC and HM wrote the paper. All authors have read and approved the final manuscript.

## Accession Numbers

Human samples: [SRA: SRP002079], adult mouse samples: [SRA: SRA001030], brain cortices samples: [SRA: SRP007262].

## Supplementary Material

Additional file 1 **this file includes figure S1 to S7**. Click here for file

Additional file 2 **this file includes supplemental data and analysis results relevant to this study, table S1 to S6, and figure legends for figure S1 to S7**. Click here for file
