# Supplementary material for: Comparative analysis of neural transcriptomes and functional implication of unannotated intronic expression
Source: BMC Genomics. 2011 Oct 10;12:494. doi: 10.1186/1471-2164-12-494 (PMC3228559; doi:10.1186/1471-2164-12-494)
Supplement: Additional file 1 — this file includes figure S1 to S7. [file 1471-2164-12-494-S1.PDF]

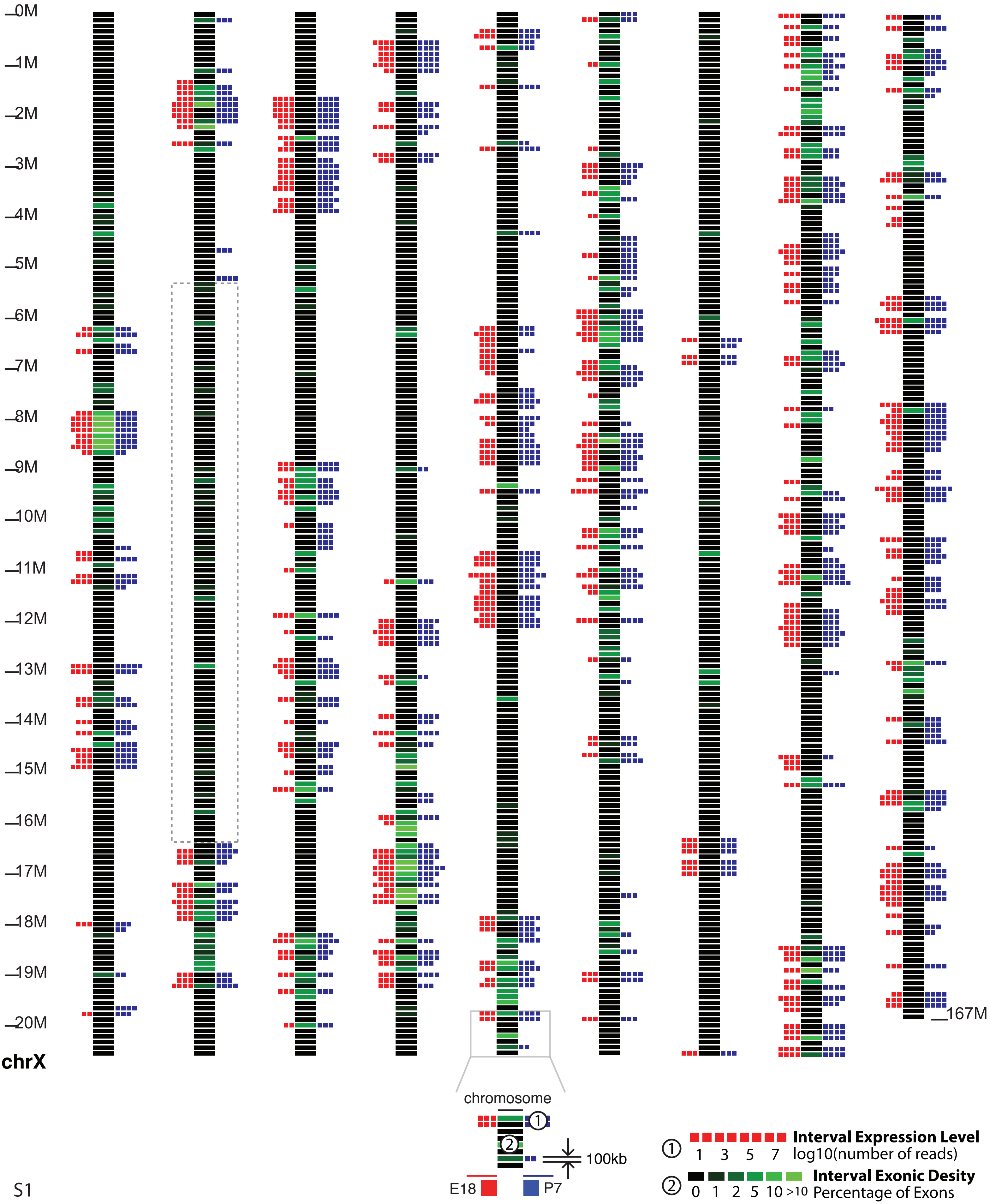

0M
1M
2M
3M
4M
5M
6M
7M
8M
9M
10M
11M
12M
13M
14M
15M
16M
17M
18M
19M
20M
167M
chrX
chromosome
①
②
100kb
E18
P7
① Interval Expression Level
1 3 5 7 log10(number of reads)
② Interval Exonic Desity
0 1 2 5 10 >10 Percentage of Exons

S1

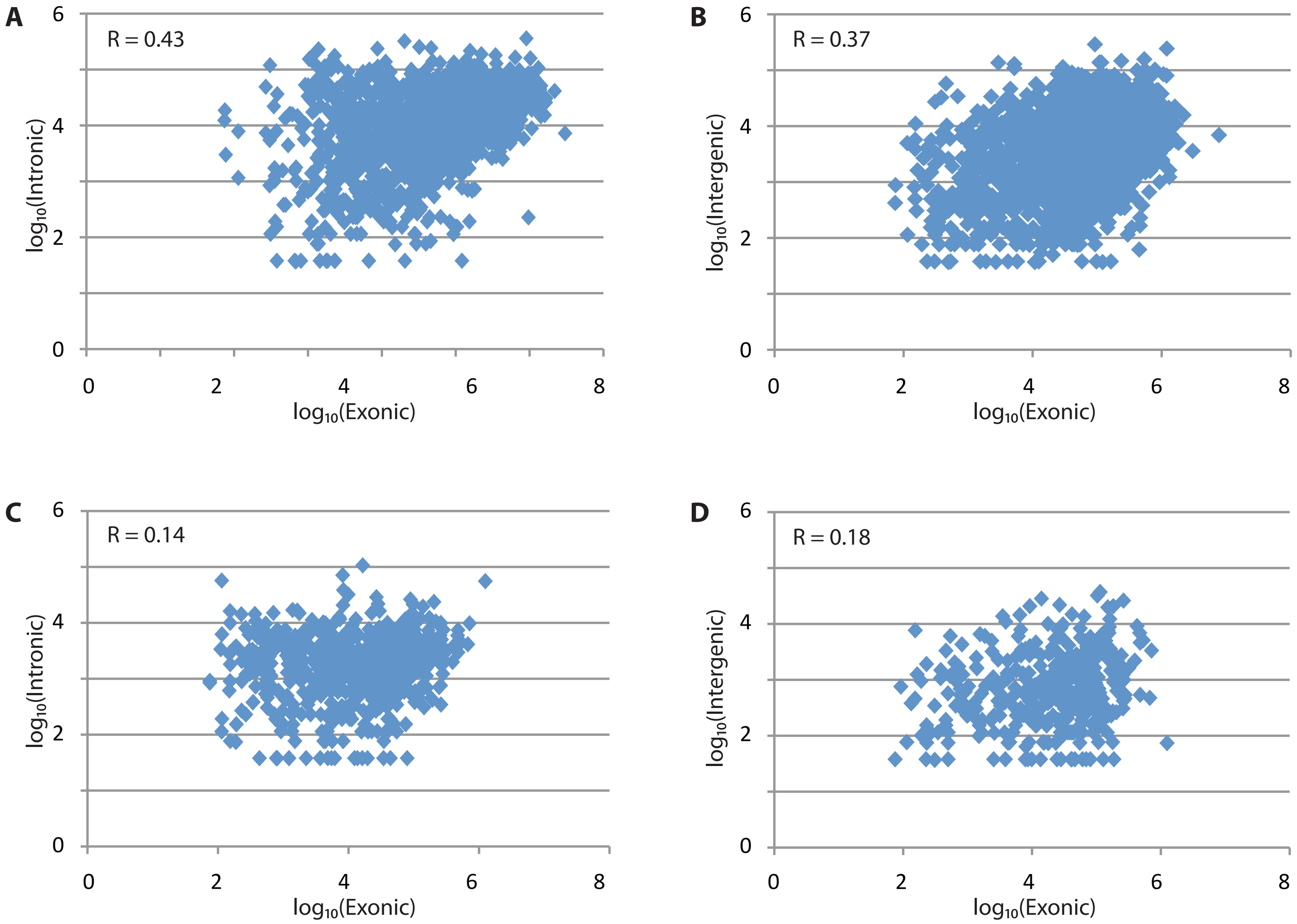
A
R = 0.43
log10(Intronic)
log10(Exonic)
B
R = 0.37
log10(Intergenic)
log10(Exonic)
C
R = 0.14
log10(Intronic)
log10(Exonic)
D
R = 0.18
log10(Intergenic)
log10(Exonic)

S2

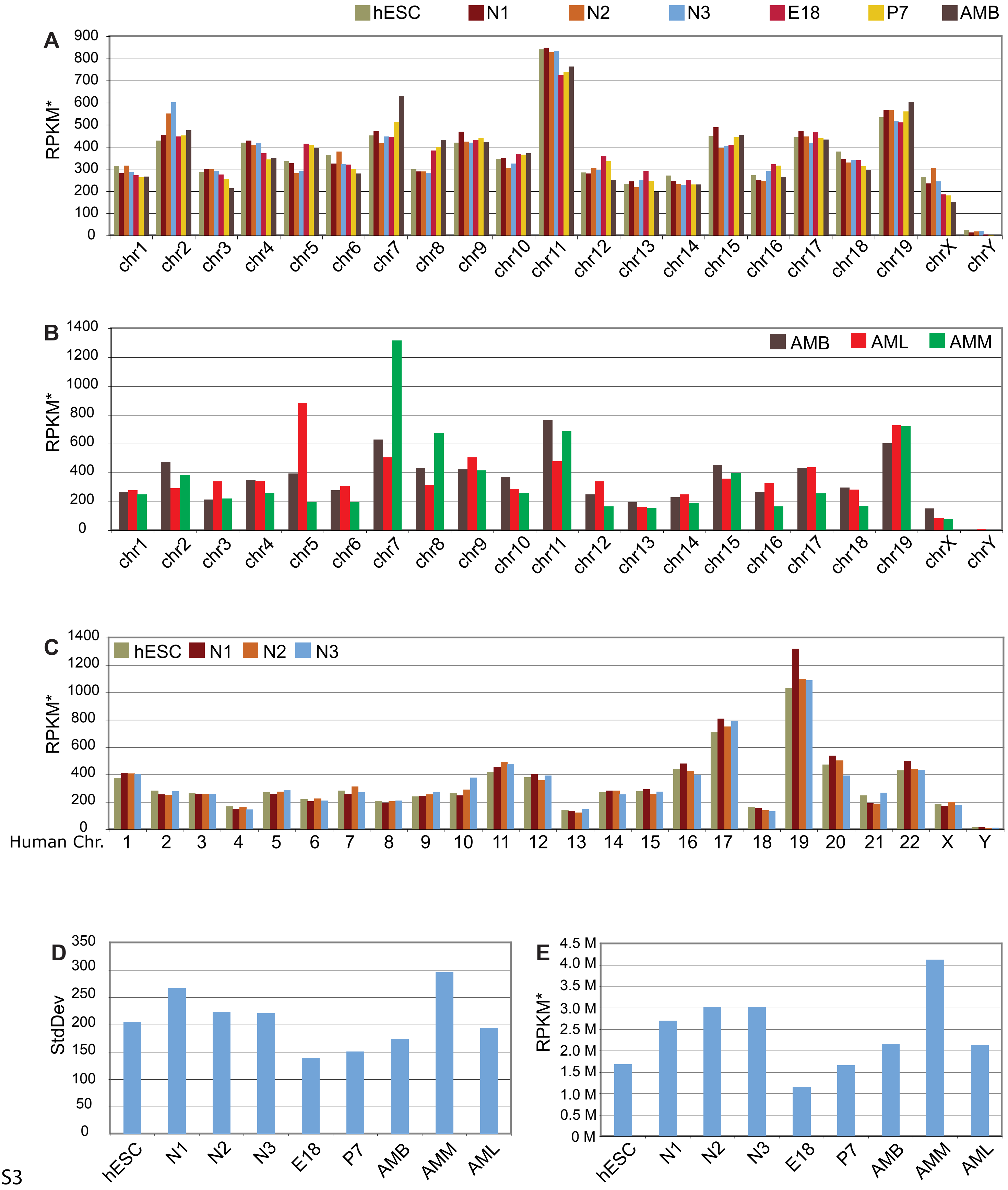
hESC
N1
N2
N3
E18
P7
AMB
A
RPKM*
0
100
200
300
400
500
600
700
800
900
chr1 chr2 chr3 chr4 chr5 chr6 chr7 chr8 chr9 chr10 chr11 chr12 chr13 chr14 chr15 chr16 chr17 chr18 chr19 chrX chrY
B
AMB
AML
AMM
RPKM*
0
200
400
600
800
1000
1200
1400
chr1 chr2 chr3 chr4 chr5 chr6 chr7 chr8 chr9 chr10 chr11 chr12 chr13 chr14 chr15 chr16 chr17 chr18 chr19 chrX chrY
C
hESC
N1
N2
N3
RPKM*
0
200
400
600
800
1000
1200
1400
Human Chr.
1 2 3 4 5 6 7 8 9 10 11 12 13 14 15 16 17 18 19 20 21 22 X Y
D
StdDev
0
50
100
150
200
250
300
350
hESC N1 N2 N3 E18 P7 AMB AMM AML
E
RPKM*
0 M
0.5 M
1.0 M
1.5 M
2.0 M
2.5 M
3.0 M
3.5 M
4.0 M
4.5 M
hESC N1 N2 N3 E18 P7 AMB AMM AML

ATP2B1

```
                                        *       920        *       940        *       960        *       980        *      1000
Human_ATP2B1b                  : ALATEPPTESLLLRKPYGRNKPLISRTMMKNILGHAFYQLVVVFTLLFAGEKFFDIDSGRNAPLHAPPSEHYTIVFNTFVLMQLFNEINARKIHGERNVF : 1000
Mouse_ATP2B1                   : ALATEPPTESLLLRKPYGRNKPLISRTMMKNILGHAFYQLVVVFTLLFAGEKFFDIDSGRNAPLHAPPSEHYTIVFNTFVLMQLFNEINARKIHGERNVF : 1000
Human_ATP2B1a                  : ALATEPPTESLLLRKPYGRNKPLISRTMMKNILGHAFYQLVVVFTLLFAGEKFFDIDSGRNAPLHAPPSEHYTIVFNTFVLMQLFNEINARKIHGERNVF : 1000
Mouse_chr10:98481907-98482067  : ---------------------------------------------------------------------------------------------------- :    -
                                 alatepptesllllrkpygrnkplisrtmmknilghafyqlvvvftllfagekffdidsgrnaplhappsehytivfntfvlmqlfneinarkihgernvf

                                        *      1020        *      1040        *      1060        *      1080        *      1100
Human_ATP2B1b                  : EGIFNNAIFCTIVLGTFVVQIIIVQFGGKPFSCSELSIEQWLWSIFLGMGTLLWGQLISTIPTSRLKFLKEAGHGTQKEEIPEEELAEDVEEIDHAEREL : 1100
Mouse_ATP2B1                   : EGIFNNAIFCTIVLGTFVVQIIIVQFGGKPFSCSELSIEQWLWSIFLGMGTLLWGQLISTIPTSRLKFLKEAGHGTQKEEIPEEELAEDVEEIDHAEREL : 1100
Human_ATP2B1a                  : EGIFNNAIFCTIVLGTFVVQIIIVQFGGKPFSCSELSIEQWLWSIFLGMGTLLWGQLISTIPTSRLKFLKEAGHGTQKEEIPEEELAEDVEEIDHAEREL : 1100
Mouse_chr10:98481907-98482067  : ---------------------------------------------------------------------------------------------------- :    -
                                 egifnnaifctivlgtfvvqiiivqfggkpfscselsieqwlwsiflgmgtllwgqlistiptsrlkflkeaghgtqkeeipeeelaedveeidhaerel

                                        *      1120        *      1140        *      1160        *      1180        *      1200
Human_ATP2B1b                  : RRGQILWFRGLNRIQTQ-----------------------------------------------------IRVVNAFRSSLYEGLEKPESRSSIHNFMTHP : 1148
Mouse_ATP2B1                   : RRGQILWFRGLNRIQTQ-----------------------------------------------------IRVVNAFRSSLYEGLEKPESRSSIHNFMTHP : 1148
Human_ATP2B1a                  : RRGQILWFRGLNRIQT-QMDVVNAFQSGSSIQGALRRQPSIASQHHDVTNISTPTHVVFSSSTASTTVGYSSGECIS---------------------- : 1176
Mouse_chr10:98481907-98482067  : -----------------QMDVVNAFQSGSSIQGALRRQPSIASQHHDVTNISTPTHVVFSSSTASTTVGC------------------------------ :   53
                                 rrgqilwfrglnriqt

                                        *      1220        *      1240        *      1260        *
Human_ATP2B1b                  : EFRIEDSEPHIPLIDDTLAEDLAPTKRNSSPPPSPNKNNNAVDSGIHLTIEMNKSATSSSPGSPLHSLETSL : 1220
Mouse_ATP2B1                   : EFRIEDSEPHIPLIDDTLAEDLAPTKRNSSPPPSPNKNNNAVDSGIHLTIEMNKSATSSSPGSPLHSLETSL : 1220
Human_ATP2B1a                  : ------------------------------------------------------------------------ :    -
Mouse_chr10:98481907-98482067  : ------------------------------------------------------------------------ :    -
```

Trim3

```
                                        *       20         *       40         *       60         *       80         *       100
Rat_1:163362070-163362170      : AGGGAGGGGACAGGAGTCCTCCAGTCAGGGCCCAGGCTGAGGGGGAACTAGAGGGGTCCTTTCCTAC-AAGAAACAGCCTCTGCAGGAAGCTTCCTGTTCAG : 101
chr7:112781296-112781396       : AGGGAGGGGACAGGAGTCCTCCAGTCAGGGCCCAGGCTAAGGGAGAACTAGAGGGGTCCTTTCCTAC-AAGACACAGCCTCTGCAGGAAGCTTCCTGTCCAG : 101
Human_chr11:6494416-6494516    : AGGGAGGGGCCTGGGGTCCTCCAGGCAGGGCTCAGGCTACAGGAGAACCAGAGGGGTCCTTTCCTACAAAAAAACAGCCTCCACATGAGGCTTTCTGTCCA- : 101
Dog_21:32977540-32977640       : AGGGAGGGGCCTGGGGTCCTCCAAGCGGGGCCCAGGCTGAAGGGGAACCAGAGGGGTCCTTTCCTAC-AACAAGCAGCCTCCGGATGAGGCTTCCCGTCCAG : 101
```

Zeb2

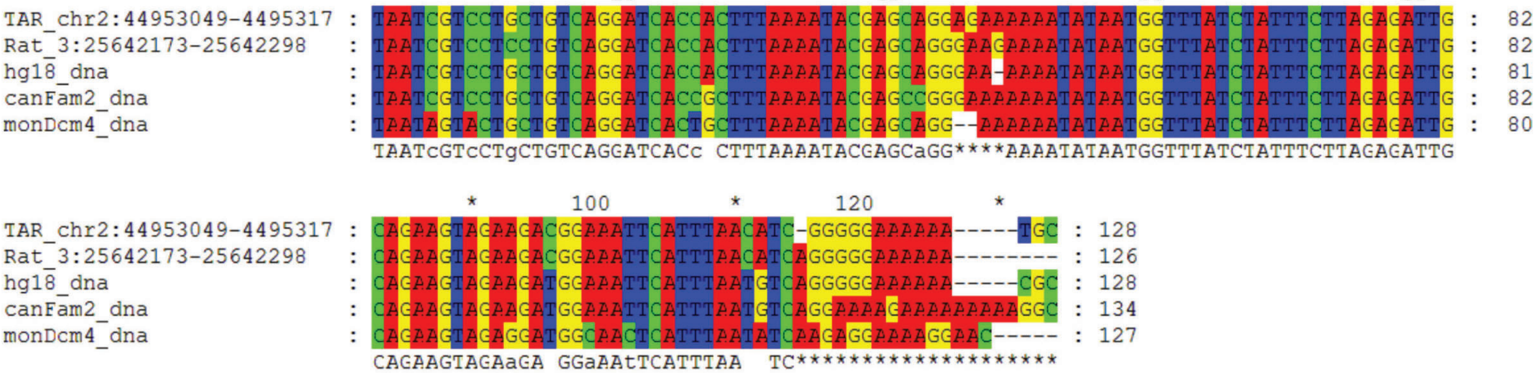

Ntrk3

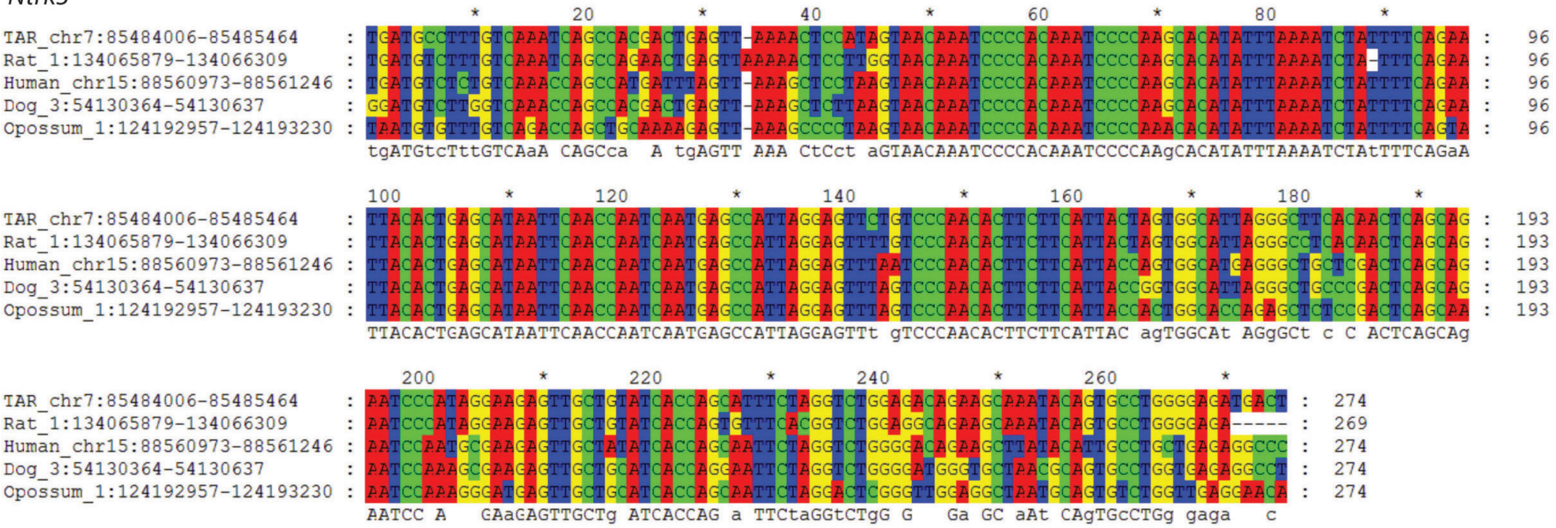

Odz2

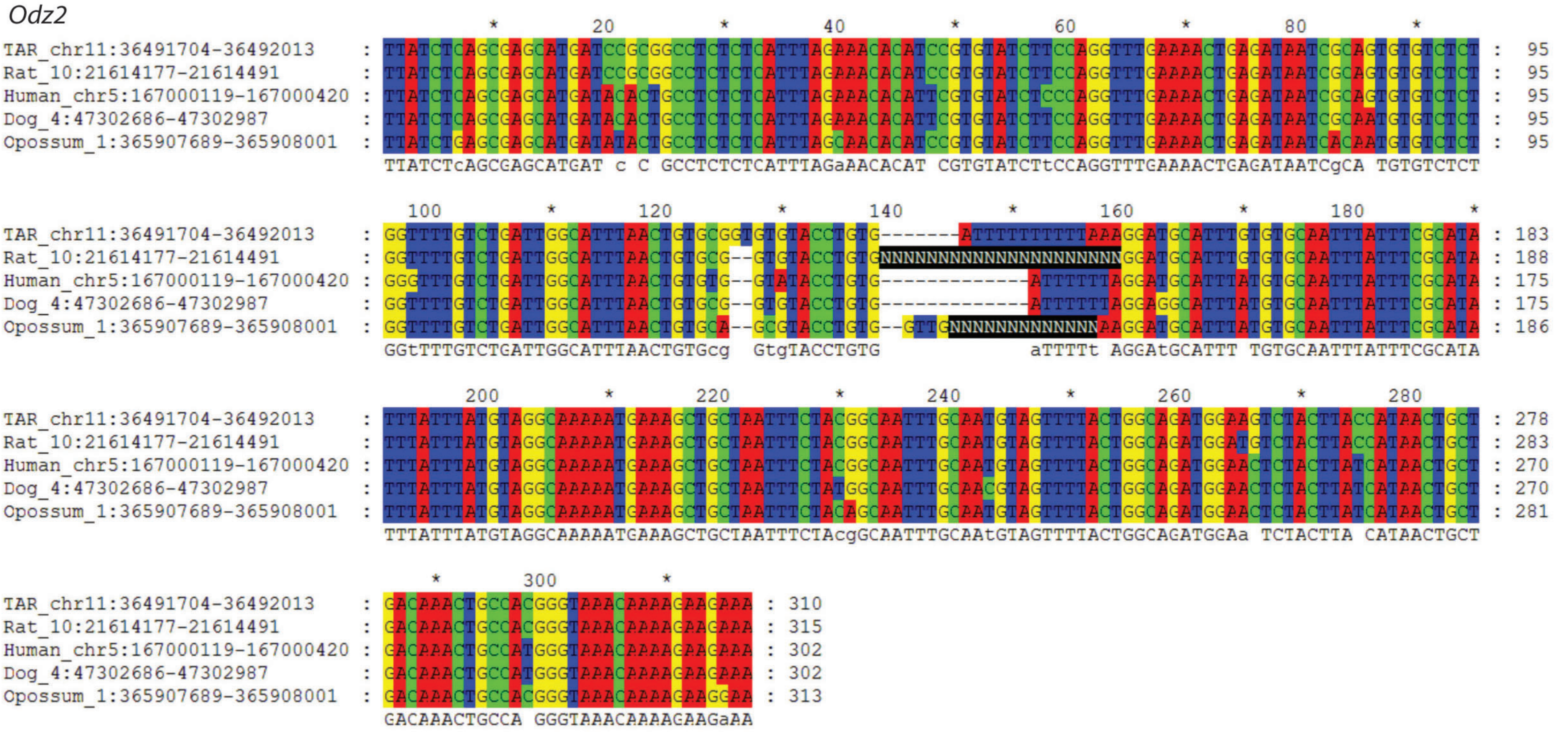

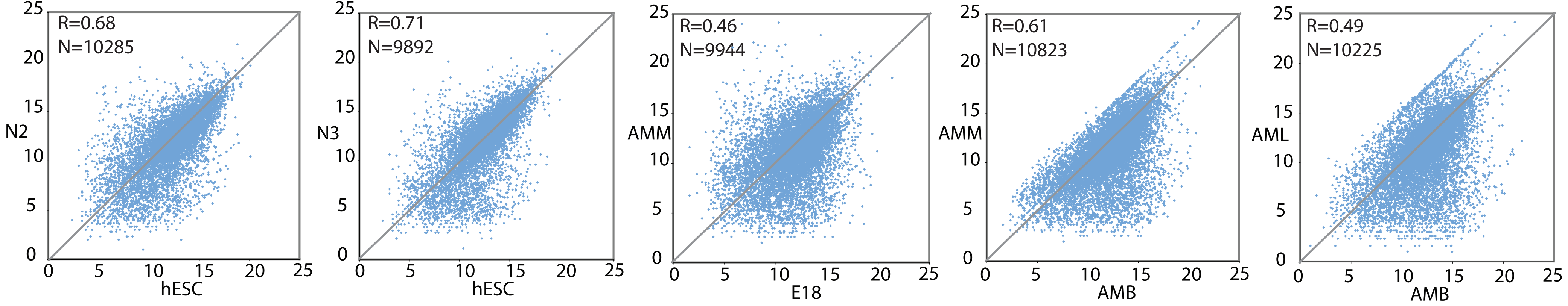

R=0.68
N=10285
N2
hESC
R=0.71
N=9892
N3
hESC
R=0.46
N=9944
AMM
E18
R=0.61
N=10823
AMM
AMB
R=0.49
N=10225
AML
AMB

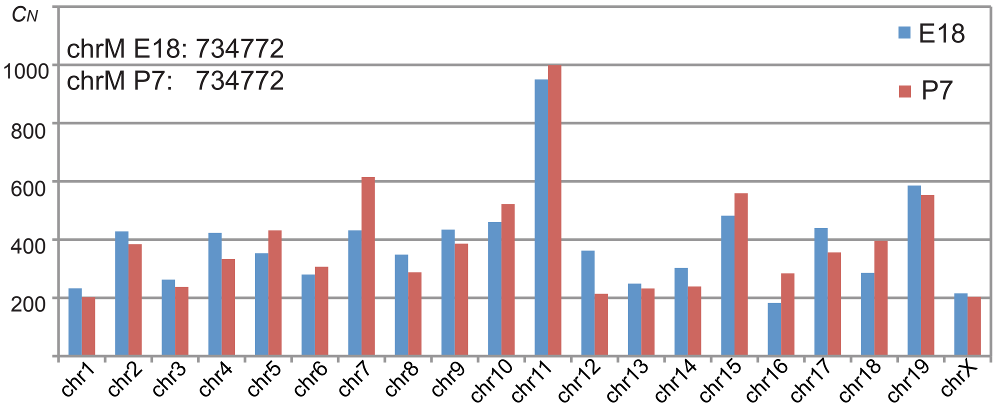

C_N
chrM E18: 734772
chrM P7: 734772
E18
P7
1000
800
600
400
200
chr1
chr2
chr3
chr4
chr5
chr6
chr7
chr8
chr9
chr10
chr11
chr12
chr13
chr14
chr15
chr16
chr17
chr18
chr19
chrX
