# Supplementary material for: Comparative analysis of neural transcriptomes and functional implication of unannotated intronic expression
Source: BMC Genomics. 2011 Oct 10;12:494. doi: 10.1186/1471-2164-12-494 (PMC3228559; doi:10.1186/1471-2164-12-494)
Supplement: Additional file 2 — this file includes supplemental data and analysis results relevant to this study, table S1 to S6, and figure legends for figure S1 to S7. [file 1471-2164-12-494-S2.DOC]

Supplemental Information

**Percentage of paired-end and single-end data mapped.**

If we allow the reads mapped to up to 10 different locations (10 hits), about 80% of the data could be mapped. Only about 15% of the data could not be mapped on the mouse reference genome. We further analyzed mapping results when we only allowed unique hit reads. For E18 stage data, about 60% of the paired-end data could be uniquely mapped onto the mm9 genome, while only about 40% of the single-end data could be uniquely mapped. For P7 stage data, the uniquely mapped percentage was 62% for paired-end data and 54% for single-end data. 57% of the entire E18 data could be uniquely mapped, and 61% of the entire P7 data could be uniquely mapped. There was no noticeable difference in reads quality between paired-end reads and single-end reads. All E18 and P7 reads, including paired-end and single-end ones, was 36 bp in length. There was no noticeable difference in reads quality between the E18 data and P7 data, either. We found that paired-end data had a higher mappability if the RNA-seq data was mapped using TopHat [1].

**The distribution of the most expressed genes among chromosomes.**

The top 500 most expressed genes were selected according to the previously published method [2]. From each chromosome, the number of genes belonging to the top 500 was counted. The counted number for each chromosome was then normalized against each chromosome’s length in a method similar to the RPKM method [3]:

|  | $C_{N}=\frac{C\times10^{12}}{C_{T}\times L}$ | (S1) |
| --- | --- | --- |

*C_N_*: normalized count for a given chromosome.

*C*: number of the most expressed genes located in the given chromosome.

*C_T_*: total number of the selected genes (in this case, 500).

*L*: length of the given chromosome (unit: bp)

The distribution of the most expressed genes among chromosomes for E18 stage was very similar to that for P7 stage (Fig. S5). Both stages’ profiles were also similar to the total gene expression distribution among chromosomes (Fig. S4).

Table S1. Chromosomal expression across stages in read counts

|  | **hESC** | **N1** | **N2** | **N3** | **E18** | **P7** | **AMB** | **AMM** | **AML** |
| --- | --- | --- | --- | --- | --- | --- | --- | --- | --- |
| **chr1** | 92476 | 89415 | 121003 | 119972 | 576642 | 617325 | 1664591 | 836121 | 1101579 |
| **chr2** | 116351 | 133228 | 195010 | 231845 | 869368 | 977243 | 2739929 | 1190394 | 1066153 |
| **chr3** | 68408 | 76658 | 92907 | 99243 | 472070 | 486040 | 1082967 | 597877 | 1091763 |
| **chr4** | 97551 | 107289 | 123988 | 137954 | 620423 | 635012 | 1724011 | 681761 | 1070897 |
| **chr5** | 76508 | 80364 | 83364 | 94414 | 676641 | 740624 | 1914836 | 503478 | 2708046 |
| **chr6** | 81063 | 78476 | 110257 | 102125 | 513719 | 538667 | 1331478 | 496872 | 926322 |
| **chr7** | 102920 | 115680 | 123946 | 144323 | 726801 | 928153 | 3047195 | 3394482 | 1550147 |
| **chr8** | 58980 | 61532 | 74119 | 79362 | 542771 | 626383 | 1802582 | 1503853 | 837867 |
| **chr9** | 77712 | 93932 | 102339 | 110617 | 574405 | 649769 | 1667401 | 874972 | 1260066 |
| **chr10** | 67252 | 73139 | 77072 | 89781 | 514335 | 565546 | 1533719 | 574747 | 755932 |
| **chr11** | 152849 | 166420 | 196167 | 215689 | 945122 | 1068548 | 2946991 | 1417059 | 1176264 |
| **chr12** | 51725 | 54751 | 71727 | 76497 | 466206 | 484446 | 968689 | 342598 | 826123 |
| **chr13** | 42278 | 47373 | 51176 | 63538 | 374994 | 351659 | 744911 | 313915 | 395946 |
| **chr14** | 50762 | 49722 | 56582 | 60894 | 335300 | 343972 | 917395 | 406472 | 631873 |
| **chr15** | 69589 | 81617 | 79826 | 88766 | 454657 | 546428 | 1489998 | 697665 | 749796 |
| **chr16** | 40058 | 39991 | 47270 | 60747 | 339079 | 369717 | 826537 | 276625 | 646429 |
| **chr17** | 63137 | 72609 | 82793 | 84524 | 475870 | 498847 | 1311107 | 413794 | 839180 |
| **chr18** | 51396 | 50382 | 58234 | 65976 | 331399 | 337969 | 855284 | 265056 | 516904 |
| **chr19** | 48964 | 55859 | 67622 | 67584 | 335801 | 408372 | 1174643 | 750516 | 899498 |
| **chrX** | 65669 | 63436 | 98576 | 86681 | 332539 | 358502 | 809519 | 223777 | 286129 |
| **chrY** | 621 | 372 | 593 | 779 | 1008 | 645 | 1033 | 1697 | 2438 |

Table S2. Principal inertias (eigenvalues) of the Correspondence Analysis

|  | **1** | **2** | **3** | **4** | **5** |
| --- | --- | --- | --- | --- | --- |
| **Value** | 0.043309 | 0.019397 | 0.001323 | 0.000912 | 0.000177 |
| **Percentage** | 66.34% | 29.71% | 2.03% | 1.4% | 0.27% |
| **Accumulated Percentage** | 66.3% | 96.1% | 98.1% | 99.5% | 99.7% |

Table S3. Manually validated expressed exons physically connected with other exons.

| Chromosome Name | Start Position | Stop Postion |
| --- | --- | --- |
| chr14 | 79688556 | 79688927 |
| chr14 | 79690050 | 79690115 |
| chr14 | 79691477 | 79691586 |
| chr7 | 25207317 | 25207797 |
| chr7 | 25206137 | 25206284 |
| chr7 | 25205830 | 25205961 |
| chr2 | 130533586 | 130535067 |
| chr16 | 66664625 | 66664753 |
| chr16 | 66661317 | 66663459 |
| chr16 | 7353023 | 7353902 |
| chr5 | 36200474 | 36200614 |
| chr5 | 36192164 | 36192845 |
| chr12 | 81268532 | 81269469 |
| chr12 | 81269844 | 81270003 |
| chr12 | 81272714 | 81272863 |
| chr7 | 29681107 | 29681254 |
| chr7 | 29681893 | 29682074 |
| chr7 | 29677979 | 29679406 |
| chr7 | 29679519 | 29679679 |
| chr7 | 29683087 | 29683270 |
| chr5 | 121975057 | 121976421 |
| chr5 | 121975057 | 121975250 |
| chr5 | 121975670 | 121975779 |
| chr5 | 121976746 | 121976871 |
| chr5 | 121978875 | 121979001 |
| chr1 | 158738249 | 158739325 |
| chr8 | 125411214 | 125411526 |
| chr6 | 28376068 | 28376500 |
| chr6 | 28375737 | 28375866 |
| chr6 | 28374721 | 28374832 |

Table S4. Manually validated expressed single exon genes (SGEs).

| Chromosome Name | Start Position | Stop Postion |
| --- | --- | --- |
| chr12 | 17178905 | 17183331 |
| chr12 | 28020080 | 28027577 |
| chr5 | 35621214 | 35624412 |
| chr15 | 84383827 | 84388253 |
| chr2 | 53936503 | 53937963 |
| chr7 | 69493162 | 69494813 |
| chrX | 119508169 | 119510994 |
| chrX | 149932774 | 149936101 |
| chr3 | 61168428 | 61172625 |
| chr14 | 109309205 | 109313456 |
| chr12 | 8504564 | 8506791 |
| chr4 | 88364195 | 88368412 |
| chr8 | 73222847 | 73224515 |
| chr9 | 123168174 | 123169907 |

Table S5. E18 intronic TARs with hits in miRbase

| **Query** | **Query Length** | **Subject** | **Subject Length** | **BitScore** | **E-value** | **Alignment Length** | **Matched** | **Gaps** |
| --- | --- | --- | --- | --- | --- | --- | --- | --- |
| chr1-33852746-33857360 | 4615 | mmu-mir-1935 | 60 | 77.8 | 1.66E-06 | 55 | 51 | 0 |
| chr1-56989661-56990743 | 1083 | mmu-mir-1935 | 60 | 85.7 | 8.28E-08 | 51 | 49 | 0 |
| chr2-6719696-6721827 | 2132 | mmu-mir-1935 | 60 | 79.8 | 6.12E-07 | 52 | 49 | 0 |
| chr3-158158463-158160069 | 1607 | mmu-mir-1935 | 60 | 71.9 | 6.78E-06 | 60 | 54 | 0 |
| chr4-45834328-45836371 | 2044 | mmu-mir-1935 | 60 | 93.7 | 5.60E-09 | 55 | 53 | 0 |
| chr11-104173757-104179094 | 5338 | mmu-mir-1935 | 60 | 95.6 | 1.65E-08 | 60 | 57 | 0 |
| chr11-57103743-57105116 | 1374 | mmu-mir-1935 | 60 | 69.9 | 6.14E-06 | 55 | 50 | 0 |
| chr12-118489259-118490932 | 1674 | mdo-mir-153-2 | 87 | 157 | 9.24E-17 | 87 | 85 | 0 |
| chr12-68308091-68309299 | 1209 | mmu-mir-1935 | 60 | 85.7 | 8.28E-08 | 55 | 52 | 0 |
| chr14-55697945-55699546 | 1602 | mmu-mir-1935 | 60 | 69.9 | 6.14E-06 | 55 | 50 | 0 |
| chr18-43006931-43008045 | 1115 | mmu-mir-1935 | 60 | 87.7 | 6.09E-08 | 60 | 56 | 0 |
| chr19-16227992-16228349 | 358 | mmu-mir-1935 | 60 | 73.8 | 1.66E-06 | 53 | 49 | 0 |

Table S6. E18 intronic TARs with hits in lncRNAdb

| **Query** | **Query Length** | **Subject** | **Subject Length** | **BitScore** | **E-value** | **Alignment Length** | **Matched** | **Gaps** |
| --- | --- | --- | --- | --- | --- | --- | --- | --- |
| chr6-49205737-49207540 | 1804 | B2 SINE RNA | 177 | 311 | 1.22E-37 | 177 | 172 | 0 |
| chr7-75179481-75181002 | 1522 | B2 SINE RNA | 177 | 165 | 1.56E-18 | 142 | 128 | 2 |
| chr11-104173757-104179094 | 5338 | B2 SINE RNA | 177 | 301 | 7.33E-36 | 176 | 170 | 0 |
| chr11-24004772-24006928 | 2157 | B2 SINE RNA | 177 | 272 | 1.98E-32 | 177 | 168 | 1 |
| chr17-5104261-5111031 | 6771 | B2 SINE RNA | 177 | 103 | 5.58E-10 | 104 | 91 | 0 |
| chr19-40419656-40421427 | 1772 | B2 SINE RNA | 177 | 311 | 1.22E-37 | 177 | 172 | 0 |

**Supplemental Figure Legends**

Figure S1. 100 Kb resolution expression map of chromosome X. All symbols represent the same information as in Fig. 1, except each horizontal box now represents only a 100 Kb genome region.

Figure S2. Expression level correlation between exonic and non-exonic regions. **A**. Genome wide exonic expression vs. intronic expression. Interval size = 1mb. **B**. Genome wide exonic expression vs. intergenic expression. Interval size = 1mb. **C**. Chromosome 11 exonic expression vs. intronic expression. Interval size = 100kb. **D**. Chromosome 11 exonic expression vs. intergenic expression. Interval size = 100kb. Only regions with both type of expression were analyzed.

Figure S3. Individual chromosome’s expression level measured in RPKM* as described in formula (1). **A**. RNA-seq reads of all neural samples, along with hESC reads, mapped onto mouse reference genome. **B**. Comparison between adult mouse brain, liver and muscle. **C**. RNA-seq reads of four human samples mapped onto human reference genome. **D**. Standard deviation (StdDev) of chromosomal expression level across datasets. **E**. Mitochondrial expression level across datasets. (hESC: human Embryonic Stem Cell, N1: early initiation of hESC, N2: neural progenitor cell induced from hESC, N3: early glial-like cell from hESC, E18: embryonic day 18 mouse brain cortices, P7: post-natal day 7 mouse brain cortices, AMB: adult mouse brain, AMM: adult mouse muscle, AML: adult mouse liver)

Figure S4. 1. Amino acid alignment of the intronic TAR detected in mouse *ATP2B1* gene and *ATP2B1* exons from mouse and human. 2. DNA alignment of the intronic TAR detected in mouse Trim 3 gene and *Trim3* exons from rat, dog and human.

Figure S5. DNA level conservation between the intronic TARs detected in mouse *Zeb2*, *Ntrk3* and *Odz2* genes and introns from rat, dog, human and opossum of the same gene.

Figure S6. Scatter plot of orthologous gene expression level between selected stages. Genes without detectable expression were not included.

Figure S7. Chromosomal distribution of the top 500 most highly expressed genes in E18 and P7 stages. Y-axis *C_N_* was calculated as decribed in formula (S1).

Reference:

1. Trapnell C, Pachter L, Salzberg SL: **TopHat: discovering splice junctions with RNA-Seq**. *Bioinformatics* 2009, **25**:1105-1111.

2. Han X, Wu X, Chung WY, Li T, Nekrutenko A, Altman NS, Chen G, Ma H: **Transcriptome of embryonic and neonatal mouse cortex by high-throughput RNA sequencing**. *Proc Natl Acad Sci U S A* 2009, **106**:12741-12746.

3. Mortazavi A, Williams BA, McCue K, Schaeffer L, Wold B: **Mapping and quantifying mammalian transcriptomes by RNA-Seq**. *Nat Methods* 2008, **5**:621-628.
